# SUMO5, a Novel Poly-SUMO Isoform, Regulates PML Nuclear Bodies

**DOI:** 10.1038/srep26509

**Published:** 2016-05-23

**Authors:** Ya-Chen Liang, Chia-Chin Lee, Ya-Li Yao, Chien-Chen Lai, M. Lienhard Schmitz, Wen-Ming Yang

**Affiliations:** 1 Institute of Molecular Biology, National Chung Hsing University, Taichung 40227, Taiwan; 2 Department of Biotechnology, Asia University, Taichung 41354, Taiwan; 3 Institute of Biochemistry, Medical Faculty, Friedrichstrasse 24, Justus-Liebig-University, 35392 Giessen, Germany

## Abstract

Promyelocytic leukemia nuclear bodies (PML-NBs) are PML-based nuclear structures that regulate various cellular processes. SUMOylation, the process of covalently conjugating small ubiquitin-like modifiers (SUMOs), is required for both the formation and the disruption of PML-NBs. However, detailed mechanisms of how SUMOylation regulates these processes remain unknown. Here we report that SUMO5, a novel SUMO variant, mediates the growth and disruption of PML-NBs. PolySUMO5 conjugation of PML at lysine 160 facilitates recruitment of PML-NB components, which enlarges PML-NBs. SUMO5 also increases polySUMO2/3 conjugation of PML, resulting in RNF4-mediated disruption of PML-NBs. The acute promyelocytic leukemia oncoprotein PML-RARα blocks SUMO5 conjugation of PML, causing cytoplasmic displacement of PML and disruption of PML-NBs. Our work not only identifies a new member of the SUMO family but also reveals the mechanistic basis of the PML-NB life cycle in human cells.

Promyelocytic leukemia nuclear bodies (PML-NBs) are non-membrane-bound domains in the cell nucleus that regulate transcription, antiviral response, DNA repair, apoptosis, senescence, and tumor suppression^1^. PML-NBs require PML to form^2^. Mature PML-NBs also contain other components such as SP100, HIPK2 and Daxx, which depends on the cellular environment^2^,^3^,^4^ and results in heterogeneity in PML-NBs. Proper generation of PML-NBs is critical to the individual as an absence or delocalization of PML-NBs results in several pathological conditions, including polyglutamine repeat neurodegenerative diseases^5^ and acute promyelocytic leukemia (APL)^6^.

Arsenic trioxide (ATO) induces the formation of PML-NBs through a two-step process: first, oxidized PML dimerizes using the BRCC domain^7^,^8^ to form a ring-like structure of PML shells. This step has been referred to as nucleation of PML-NBs^9^. PML shells then start to mature by recruiting additional PML-NB components^1^. In cells without oxidative stress, PML-NBs become prominent when cells enter G1 phase. During mitosis, PML-NBs fall apart and PML forms aggregates. PML-NB components start to enter the bodies to colocalize with PML after mitotic exit^10^,^11^, suggesting that the initiation of PML-NB formation happens at the end of mitosis. Taken together, current evidence suggests that the biogenesis of PML-NBs, which finishes in G1, needs at least two steps: nucleation through PML dimerization and the recruitment of PML-NB components.

The recruitment of PML-NB components depends on SUMOylation^2^,^12^ as loss of UBC9, an E2 ligase for SUMOylation, blocks the formation of PML-NBs^13^. PML can be conjugated by SUMO1 on three lysines: K65, K160, and K490^12^,^14^,^15^,^16^,^17^. PML also contains a SUMO interaction domain (SIM), which mediates interaction with SUMOs. As PML-NB components are also SUMO1-conjugated or SIM-containing proteins^18^, it was proposed that PML-NBs mature through SUMO-SIM interaction networks that recruit PML-NB components, causing PML shells to enlarge^19^. However, a recent report showing that loss of SUMO1 does not block the formation of NBs suggests the formation of PML-NB requires more than SUMO1 conjugation of PML^20^.

Similar to the formation of PML-NBs, disruption of PML-NBs also requires SUMOylation. Under oxidative stress, PML is conjugated by polySUMO2/3 chains^21^. RNF4, a ubiquitin ligase, is recruited to interact with SUMO2/3-conjugated PML using its SIM domain^22^. RNF4 then polyubiquitinates the PML-NB components^17^, resulting in their degradation in proteasomes and the disruption of PML-NBs.

In addition to oxidative stress, PML-NBs can be disrupted by oncoprotein PML-RARα^23^,^24^,^25^,^26^, which is produced as a result of gene fusion between *PML* and *RARα* in APL patients. Although it is not clear how PML-RARα causes the disruption of PML-NBs, several clues suggest that SUMOylation of K160 was involved in this process: PML-RARα is a SUMOylated protein^14^, and SUMOylation at K160 of PML-RARα is critical for leukemic transformation^27^. Moreover, during RNF4-mediated disruption of PML-NBs, polySUMO2/3 conjugation of PML also takes place at K160^28^. Interestingly, K160 has also been suggested to be the key lysine for NB formation^14^,^15^,^16^. Taken together, K160 seems to be critical to both the biogenesis and the disruption of PML-NBs. However, how PML K160 mediates these processes is not clear either.

To understand how PML-NBs form in the absence of SUMO1, how K160 of PML controls the life cycle of PML-NBs, and how SUMOylation is involved in PML-RARα-mediated disruption of PML-NBs, we looked for other SUMO isoforms that could regulate PML-NBs. Here, we report the identification of SUMO5 that regulates the formation and disruption of PML-NBs in human cells. PolySUMO5 conjugation on K160 of PML results in recruitment of proteins to form PML-NBs. SUMO5 conjugation on PML is gradually replaced by SUMO2/3 conjugation, which leads to RNF4-mediated disruption of PML-NBs. Furthermore, PML-RARα causes disruption of PML-NBs by depriving PML of SUMO5 conjugation, leading to cytoplasmic displacement of PML. Our findings demonstrate that SUMO5 is a natural link controlling the dynamics of PML-NBs.

## Results

### SUMO5 is a novel, primate-specific and tissue-specific small ubiquitin-related modifier

Using total RNA and primers based on the nucleotide sequence of *SUMO1*, we cloned a novel SUMO isoform we called *SUMO5*. The *SUMO5* gene (GenBank: FJ042790.1) is located on chromosome 20 while *SUMO1* (GenBank: NG_011679.1) is located on chromosome 2 (2q33). The cDNA of human *SUMO5* contains a protein-coding sequence of 306 nucleotides, a 3′ noncoding region, and a poly(A) tail signal (Fig. S1A).

**Figure 1:**
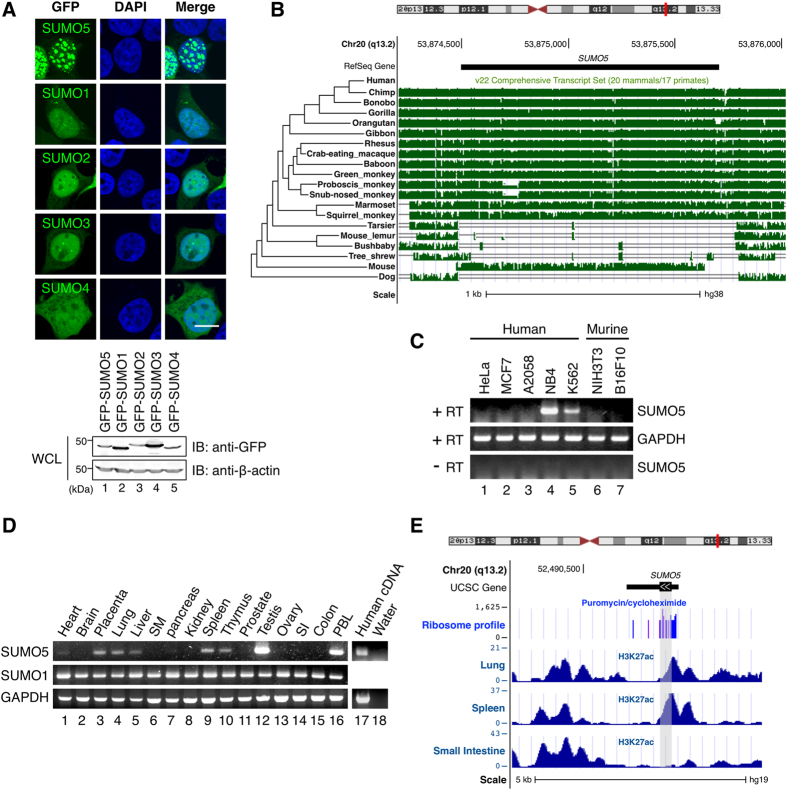
A primate- and tissue-specific SUMO variant, SUMO5, forms NBs. (**A**) SUMO5 forms NBs. (Top panel) localization of GFP-tagged SUMO proteins and their associated subcellular structures in 293 cells were assessed by confocal microscopy. DNA (blue) was stained with DAPI. Scale bar, 10 μm. (Bottom panel) whole cell lysates were immunoblotted with anti-GFP or anti-β-actin antibodies. (**B**) UCSC Genome Browser display of Multiz Alignments of 20 mammals (17 primates) at the *SUMO5* gene locus (chr20:53, 874, 198-53, 876, 011), indicating SUMO5 is conserved among primate species. The height of species track (green histogram) reflects the value of normalized MULTIZ score^46^. Organisms (species) included are: Human (*Homo sapiens*), Chimp (*Pan troglodytes*), Bonobo (*Pan paniscus*), Gorilla (*Gorilla gorilla gorilla*), Orangutan (*Pongo pygmaeus abelii*), Gibbon (*Nomascus leucogenys*), Proboscis monkey (*Nasalis larvatus*), Golden snub-nosed monkey (*Rhinopithecus roxellana*), Green monkey (*Chlorocebus sabaeus*), Crab-eating macaque (*Macaca fascicularis*), Rhesus (*Macaca mulatta*), Baboon (*Papio anubis*), Squirrel monkey (*Saimiri boliviensis*), Marmoset (*Callithrix jacchus*), Tarsier (*Tarsius syrichta*), Mouse lemur (*Microcebus murinus*), Bushbaby (*Otolemur garnettii*), Tree shrew (*Tupaia belangeri*), Dog (*Canis lupus familiaris*), and Mouse (*Mus musculus*). Their phylogenetic relationship is listed at the left of the panel. (**C**) SUMO5 is expressed in human cell lines. Expression of SUMO5 mRNA was analyzed by RT-PCR. (**D**) SUMO5 expression is tissue-specific. mRNA levels of SUMO5 and SUMO1 were analyzed by RT-PCR. SM, skeletal muscle. SI, small intestine. PBL, peripheral blood leukocyte. (**E**) UCSC Genome Browser display of translation initiation sites and histone H3 K27 acetylation marks at the putative *SUMO5* promoter region. (Ribosome profile) Translation initiation sites were indicated by ribosome profiles of THP-1 cells treated with puromycin (blue) and with cycloheximide (purple) as control. The ribosome profiles data and track were generated as described^31^,^32^. The height of the histogram at each location corresponds to the number of reads. A vertical, gray block indicates the coding region of *SUMO5*. (Bottom three tracks) *SUMO5* is transcribed in the lung and the spleen. H3 K27 acetylation marks, which indicate actively transcribed genes, were mapped using ChIP-Seq by the Roadmap Epigenomics Project for the lung (GEO Accession: GSM906395), spleen (GSM906398), and small intestine (GSM915330).

To understand the biological functions of SUMO5, first we followed previous studies using exogenously expressed SUMO isoforms^17^,^19^,^29^,^30^ to compared their subcellular localization in 293 cells. We found that SUMO5 formed prominent non-membrane-bound structures in the nucleus better than SUMO1-4 did (Fig. 1A, upper panel). All SUMO isoforms expressed to comparable levels, excluding the possibility that different levels of expression caused variations in the ability to form nuclear bodies (NBs) (Fig. 1A, bottom panel).

Next, by further blasting non-redundant protein sequence (nr) databases, we found that SUMO5 was highly conserved among 17 primate species but more distantly related to the mouse ortholog (Fig. 1B, compare height of histograms). The expression of *SUMO5* was also detected by RT-PCR in NB4 and K562, two human leukemia cell lines, but not in cells from other human tissues such as HeLa, MCF7, A2058 or from mice (Fig. 1C). *SUMO5* exhibited strict tissue specificity, with exceptionally high expression levels in testes and peripheral blood leukocytes (PBL) (Fig. 1D). In contrast, *SUMO1* expression was detected in all tissues examined (Figs 1D and S1B,C), with the expression of *SUMO1* exceeded that of *SUMO5* in the same tissue (Fig. S1D). Moreover, genome-wide ribosome profiling and ChIP-seq data showed that translation initiation sites and histone H3 lysine 27 acetylation (H3K27ac), an active promoter marker, were found around the 5′ position of the *SUMO5* coding region (Fig. 1E), indicating *SUMO5* is translated in human lung and spleen tissues^31^,^32^. These results demonstrated that SUMO5 was a novel, tissue-specific member of the SUMO family that’s highly conserved among primate species.

### SUMO5 induces NB formation by conjugation, which requires K18

**Figure 2:**
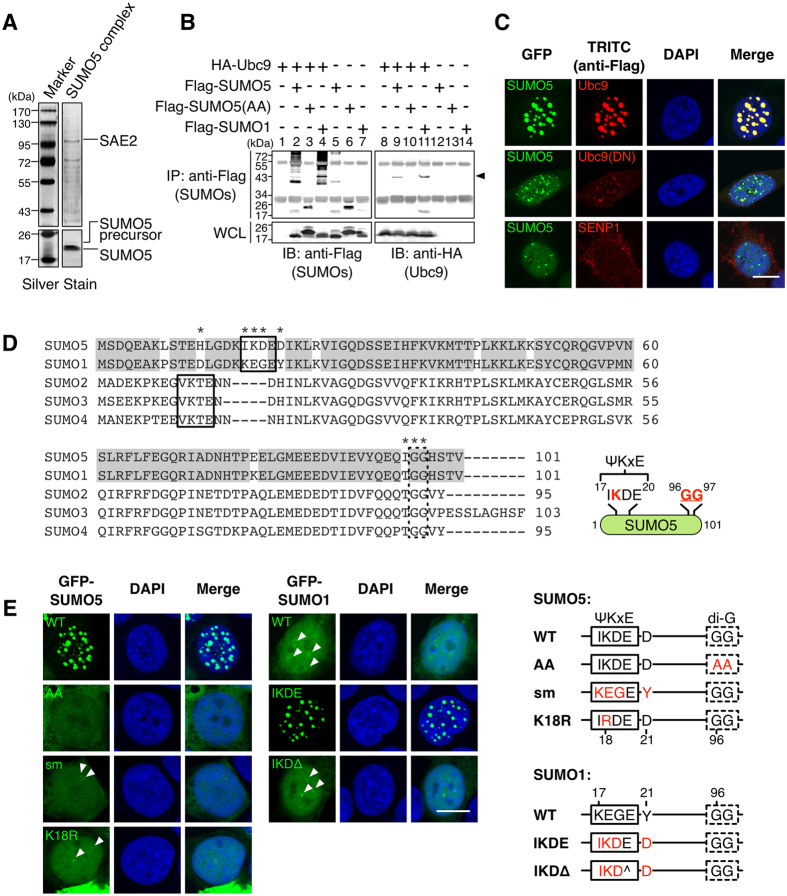
SUMO5 forms NBs through SUMO conjugation. (**A**) SAE2, a SUMO E1 enzyme, is a member of the SUMO5 protein complex. Lysates from 293 cells expressing Flag-SUMO5 were purified by an immunoaffinity column and analyzed by 8% and 12% SDS-PAGE. Protein bands specific to the SUMO5 complex were identified by mass spectrometry. (**B**) Ubc9 is a SUMO E2 enzyme for SUMO5. 293 cells were transfected with expression plasmids as indicated. Anti-Flag immunoprecipitates of cell lysates were immunoblotted with anti-Flag or anti-HA antibodies. Arrowhead indicates SUMO-conjugated Ubc9. (**C**) Ubc(DN) and SENP1 block the formation of SUMO5 NBs. NBs in 293 cells co-expressing GFP-SUMO5 and Flag-tagged Ubc9, Ubc9(DN), or SENP1 were visualized by confocal microscopy. (**D**) Alignment of amino acid sequences of SUMO5, SUMO1, SUMO2, SUMO3, and SUMO4. Conserved SUMO conjugation motifs are boxed by solid lines. The di-glycine motif whose removal is required for SUMO activation is boxed by dashed lines. *indicates amino acid residues in SUMO5 that were point-mutated in this study. Identical amino acids between SUMO5 and SUMO1 are shaded. Sequence alignment was performed using ClustalW. Diagram at lower right shows the conserved SUMO modification motif and the Gly-Gly conjugation site of SUMO5. (**E**) SUMO5 forms NBs through polySUMOylation at K18. 293 cells were transfected with expression plasmids as indicated and analyzed by confocal microscopy. The locations of mutations are listed in the right panel. Arrowheads indicate the positions of small PML-NBs. Scale bars, 10 μm.

To determine if SUMO5 was capable of SUMO conjugation (SUMOylation), we tested if SUMO5 interacted with proteins involved in the SUMOylation process. SUMOylation requires the collaboration of SUMO E1, E2, and sometimes E3 enzymes, whereas attached SUMOs can be deconjugated by SUMO proteases, SENPs. Using an anti-Flag immunoaffinity purification strategy, we found that SAE2, a SUMO E1 enzyme, was present in the SUMO5 protein complex (Fig. 2A). Co-immunoprecipitation showed that Ubc9, the SUMO E2 enzyme, was covalently linked to SUMO5 (Fig. 2B, lanes 2 and 9). SUMO5(AA), a mutant whose C-terminal Gly-Gly motif required for SUMO conjugation were mutated to Ala-Ala, lost the ability to conjugate Ubc9 (Fig. 2B, lanes 3 and 10). Co-IP screening also revealed that SUMO5 specifically interacted with SUMO E3 enzymes Pc2 and PIAS1, but not with PIASx-α (Fig. S2A). More important, the formation of SUMO5-induced NBs was impaired by coexpression of Ubc9(DN), a dominant negative mutant of Ubc9, or SENP1, the SUMO de-conjugating protease (Fig. 2C). These results suggest that SUMO5 is capable of conjugation, and conjugation is required for the formation of NBs.

Sequence analysis showed that SUMO5 contained a conserved SUMO modification motif IKDED (residues 17–21). This motif is absent in SUMO1 but corresponds in position to the SUMO modification motif of SUMO2/3 (Figs 2D and S2B). To test whether the putative SUMO modification motif of SUMO5 is required for the formation of NBs, we created the following amino acid-substitution mutants: SUMO5(sm), SUMO5(K18R), SUMO1(IKDE), SUMO5(H12D), SUMO5(I95T) and a compound mutant SUMO1(IKDΔ). In SUMO5(sm), IKDED were changed into KEGEY, creating a SUMO5 protein in which residues 17–21 were from SUMO1. In SUMO5(K18R), the acceptor lysine in the SUMO modification motif was mutated to arginine. In SUMO1(IKDE), KEGEY were changed into IKDED, forming a SUMO1 with the SUMO modification motif from SUMO5. In SUMO1(IKDΔ), KEGEY were mutated and shortened into IKDD (Fig. 2E, right panel).

Confocal microscopic analysis of cells expressing these mutant SUMO proteins (Fig. 2E) showed that: (1), residues 17–21 in SUMO5 (IKDED) were required for NB formation, as SUMO5(sm) could not form NBs while SUMO1(IKDE) could; (2), both K18 and the di-glycine conjugation motif of SUMO5 were required to form NBs, as SUMO5(K18R) and SUMO5(AA) were unable to form NBs; (3), the formation of NBs required the complete IKDED sequence, as SUMO1(IKDΔ) was unable to form NBs. SUMO5(H12D) and SUMO5(I95T) also formed NBs (Fig. S2C), suggesting that although SUMO5 and SUMO1 differed at residues 12 and 95, these residues were not critical regarding NB formation. Moreover, SUMO5(sm) was dominant negative over SUMO5 as co-expression of SUMO5(sm) and SUMO5 resulted in a loss of SUMO5-induced NBs (Fig. S2D). These results show that SUMO5-induced NB formation requires the complete IKDED sequence that is absent in SUMO1 as well as the C-terminal di-glycine conjugation motif.

### SUMO5 facilitates PML-NB formation

**Figure 3:**
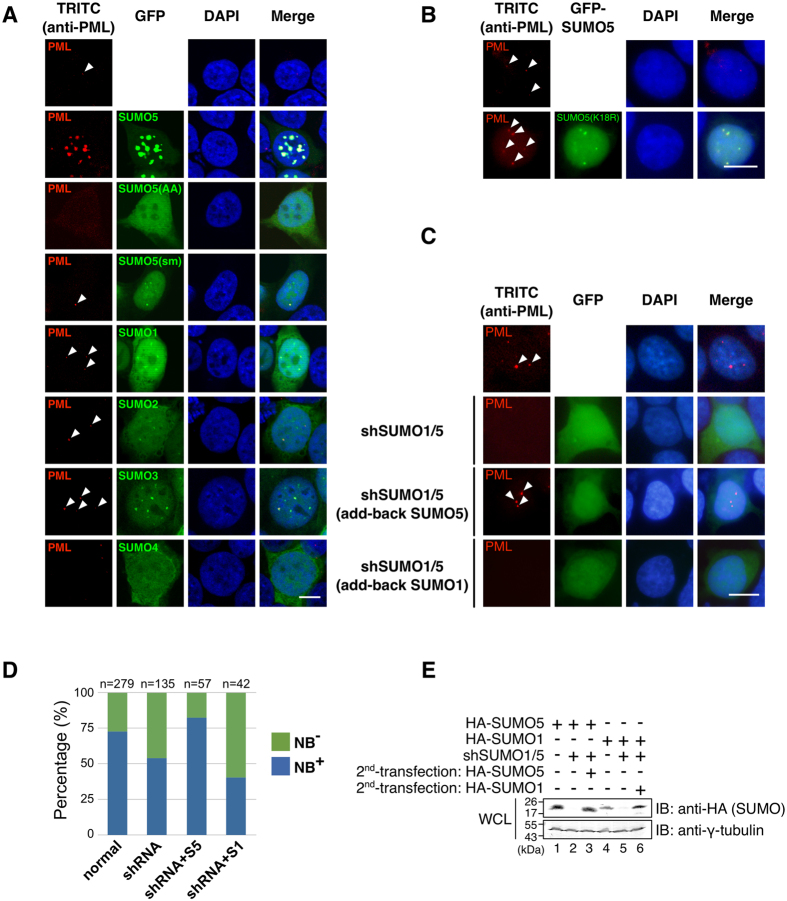
SUMO5 facilities the formation of PML-NBs. (**A**) The conjugation site and the SUMO modification motif of SUMO5 are required for the formation and the enlargement of PML-NBs, respectively. GFP-tagged SUMO5, SUMO5(AA), SUMO5(sm), SUMO1, SUMO2, SUMO3, and SUMO4 were transfected into 293 cells. The formation of endogenous PML-NBs (red) was assessed by confocal microscopy using an anti-PML antibody. Arrowheads indicate the positions of small PML-NBs. All of the cells expressing GFP-SUMO5, -SUMO5(sm), -SUMO1, -SUMO2, or -SUMO3 contained NBs, and representative images are shown. None of the cells expressing GFP-SUMO4 contained NBs. 8% of the cells expressing GFP-SUMO5(AA) contained detectable NBs. (**B**) SUMO5(K18R) forms small yet detectable PML-NBs. 293 cells were transfected with GFP-tagged SUMO5(K18R) and analyzed as in (**A**). Arrowheads indicate the positions of PML-NBs. (**C**) SUMO5 restores PML-NBs that had been demolished by SUMO shRNA. 293 cells were transfected with an shRNA construct targeting both SUMO1 and SUMO5 (shSUMO1/5) and then re-transfected with HA-SUMO5 or HA-SUMO1 24 hours later. Restoration of endogenous PML-NBs (red) was assessed by immunofluorescence microscopy with anti-PML antibody and indicated by arrowheads. Expression of SUMO1/5 shRNA was verified by GFP fluorescence. (**D**) The percentages of GFP-expressing cells from (**C**) with or without PML-NBs were calculated. “n” represents the total number of cells counted. In untransfected cells, 73% had detectable NBs (NB+). In shRNA-treated (shRNA) cells, 54% were NB+. In SUMO5-rescued cells (shRNA + S5), 82% were NB+. In SUMO1-rescued cells (shRNA+S1), 40% were NB+. (**E**) Adding back SUMO1 or SUMO5 restores expression after shRNA knockdown. 293 cells were treated as in (**C**). Whole cell lysates were immunoblotted with anti-HA or anti-γ-tubulin antibodies. Scale bars, 10 μm.

As SUMO5-induced NBs morphologically resembled PML-NBs, we hypothesized that SUMO5 regulates the formation of PML-NBs. Endogenous PML-NBs in cells were few and small (Fig. 3A, top panels). Ectopic expression of SUMO1, 2, or 3 slightly increased the number of PML-NBs but did not alter their small size. Expression of SUMO5, however, increased both the number and the size of PML-NBs. Cells expressing conjugation deficient SUMOs, such as SUMO4 or SUMO5(AA), lacked detectable PML-NBs. On the other hand, cells expressing SUMO5(sm) or SUMO5(K18R) contained small PML-NBs, similar to the effect of the expression of SUMO1 (Fig. 3A,B).

To further distinguish the functions of SUMO5 and SUMO1 regarding the control of PML-NB formation, we created a cell-based reconstitution system by depleting both SUMO1 and SUMO5 using an shRNA construct and then adding back SUMO5 or SUMO1. The percentage of cells having detectable PML-NBs decreased from 73% to 54% when both SUMO1 and SUMO5 were knocked down (Figs 3C–E and S3). When SUMO5 was added back, 82% of the cells had PML-NBs restored (Fig. 3C,D). However, when SUMO1 was added back, PML-NBs did not restore (Fig. 3C,D, 40% of cells with SUMO1 added back contained PML-NBs). This result revealed that SUMO5 and SUMO1 were not functionally redundant. In summary, SUMO5 that is conjugation-competent (with the di-glycines) and contains the SUMO modification motif (IKDED) can increase both the number and the size of PML-NBs.

### SUMO5 forms polymeric chains on K160 of PML

**Figure 4:**
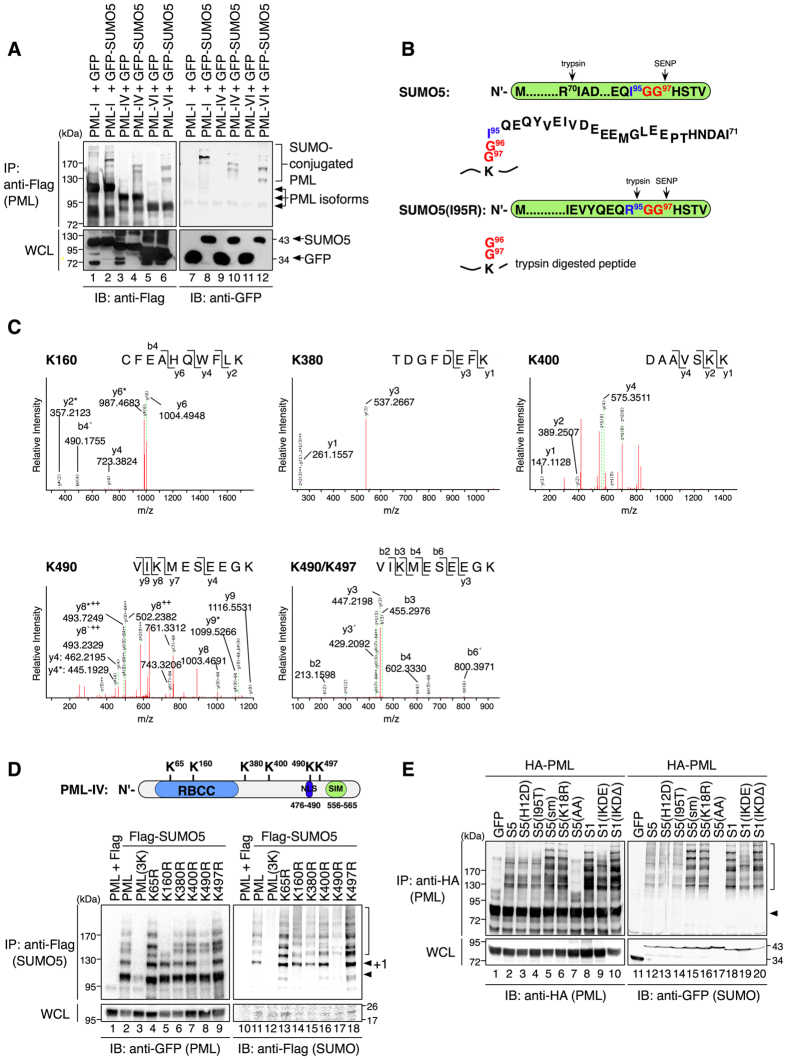
SUMO5 conjugates PML as polymeric chains. (**A**) SUMO5 can conjugate PML. GFP-tagged SUMO5 was co-expressed with Flag-tagged PML-I, PML-IV, or PML-VI in 293 cells. Anti-Flag immunoprecipitates were immunoblotted with anti-Flag or anti-GFP antibodies as indicated. (**B**) SUMO5(I95R), which retains the di-glycine motif (G96G97) after tryptic digest, is used to identify peptide fragments conjugated by SUMO5 in LC-MS/MS analyses. (**C**) MS/MS confirms that SUMO5 conjugates PML at K160, K380, K400, K490, K497, and K490/K497. Anti-Flag immunoprecipitates from 293 cell lysates expressing Flag-PML-IV and GFP-SUMO5(I95R) were separated by SDS-PAGE and analyzed by LC-MS/MS. (**D**) SUMO5 conjugates PML at K65, K160, and K490. Flag-tagged SUMO5 was co-expressed with GFP-tagged wild-type or mutant PML. Anti-Flag immunoprecipitates were immunoblotted with anti-GFP or anti-Flag antibodies. PML(3K), mutant PML with lysines 65, 160, and 490 mutated to arginines. Bracket, SUMO-modified PML. Arrowhead, unmodified PML. +1 next to an arrowhead indicates the position of PML conjugated by one SUMO5 molecule. (**E**) SUMO5 poly-SUMOylates PML. GFP-tagged SUMO5 or mutants were co-expressed with HA-tagged PML in 293 cells. Anti-HA immunoprecipitates were immunoblotted with anti-HA or anti-GFP antibodies as indicated. Bracket, SUMO-modified PML. Arrowhead, unmodified PML.

We hypothesized that SUMO5 increased the size and number of PML-NBs through conjugation of PML. By detecting SUMO conjugation on PML using immunoprecipitation from cell lysates, we confirmed that PML-I, -IV and -VI, three PML isoforms we examined, were conjugated by SUMO5 (Fig. 4A). Moreover, conjugation of SUMO5 to PML was mediated by Ubc9 (Fig. S4A) and deconjugation was mediated by SENP1 (Fig. S4B, lanes 2 and 4). To identify SUMO5 conjugation sites on PML, we generated a SUMO5 mutant, SUMO5(I95R), with a strategically located tryptic cleavage site at the C-terminus to facilitate the recovery and identification of SUMOylated peptides in LC-MS/MS analysis (Fig. 4B). We co-transfected Flag-PML-IV and GFP-SUMO5(I95R) in 293 cells, immunoprecipitated PML, and analyzed SUMO5 conjugation products by polyacrylamide gel electrophoresis, silver staining, and LC-MS/MS. As shown in Figs 4C and S4C, SUMO5 conjugated PML at lysine 160 (K160), K380, K400, K490, and K497.

Next, we examined whether different SUMO5 conjugation sites on PML were functionally distinct by using PML point mutants (Fig. 4D). PML(3K), a mutant with lysines 65, 160, and 490 mutated to arginines 14, completely lost the ability to be conjugated by SUMO5 (Fig. 4D, lanes 3 and 12) but could be conjugated by SUMO1 (Fig. S4D). On the other hand, K380R, K400R, and K497R mutants shared the same conjugation patterns with PML (Fig. 4D, compare lanes 6, 7, 9 to lane 2), suggesting that these lysines were not key acceptor lysines for SUMO5 conjugation or compensatory conjugations occurred as single lysines were mutated^33^. Single K to R mutation at residue 490 abolished conjugation of one SUMO5 to PML (Fig. 4D, lane 8), indicating that SUMO5 mono-conjugated K490. PML K65R mutant was completely conjugation-competent, confirming that K65 was not conjugated by SUMO5 (Fig. 4D, lane 4). K160R mutation of PML compromised the formation of high molecular weight species of SUMO5 conjugation on PML, suggesting that PML was conjugated by multiple molecules of SUMO5 at K160 (Fig. 4D, lane 5).

SUMO5 contained an internal SUMO modification motif (IKDED) required for PML-NB formation (Fig. 3). Through LC-MS/MS analysis, we confirmed that SUMO5 self-conjugated at K18 within the IKDED motif (Fig. S4E). SUMO5, SUMO5(H12D) and SUMO5(I95T), which all retained K18, created high molecular-weight species on PML, indicating they were capable of conjugating PML as polymers (Fig. 4E, lanes 2, 3, and 4). High-molecular-weight bands of PML created by these SUMO5 proteins appeared as smearing, which corresponded to heterogeneous SUMO polymers containing combinations of GFP-SUMO5 and endogenous SUMO5 or SUMO2/3^15^.

Although PML conjugated by wild-type SUMO5 appeared as smearing, conjugation by SUMO5(sm), SUMO5(K18R) or SUMO1 created distinct SUMOylated species of PML as sharp ladders (Fig. 4E, lanes 5, 6 and 8; also see lanes 15, 16, and 18 for comparison), which represented PML conjugated by monomers of GFP-SUMOs. More important, SUMO1(IKDE) created patterns of SUMO conjugation similar to SUMO5 did (Fig. 4E, lanes 9 and 19), while SUMO1(IKDΔ) created ladders of SUMO conjugation similar to SUMO5(sm) (Fig. 4E, lanes 10 and 20), confirming that SUMO5 self-conjugates at K18 within the SUMO modification motif. SUMO5(AA) failed to conjugate PML as expected (Fig. 4E, lanes 7 and 17). These results confirm that SUMO5 forms polymeric chains on K160 of PML by successive conjugation at K18.

### SUMO5 conjugation to PML leads to recruitment of PML-NB components and enlargement of PML-NBs

**Figure 5:**
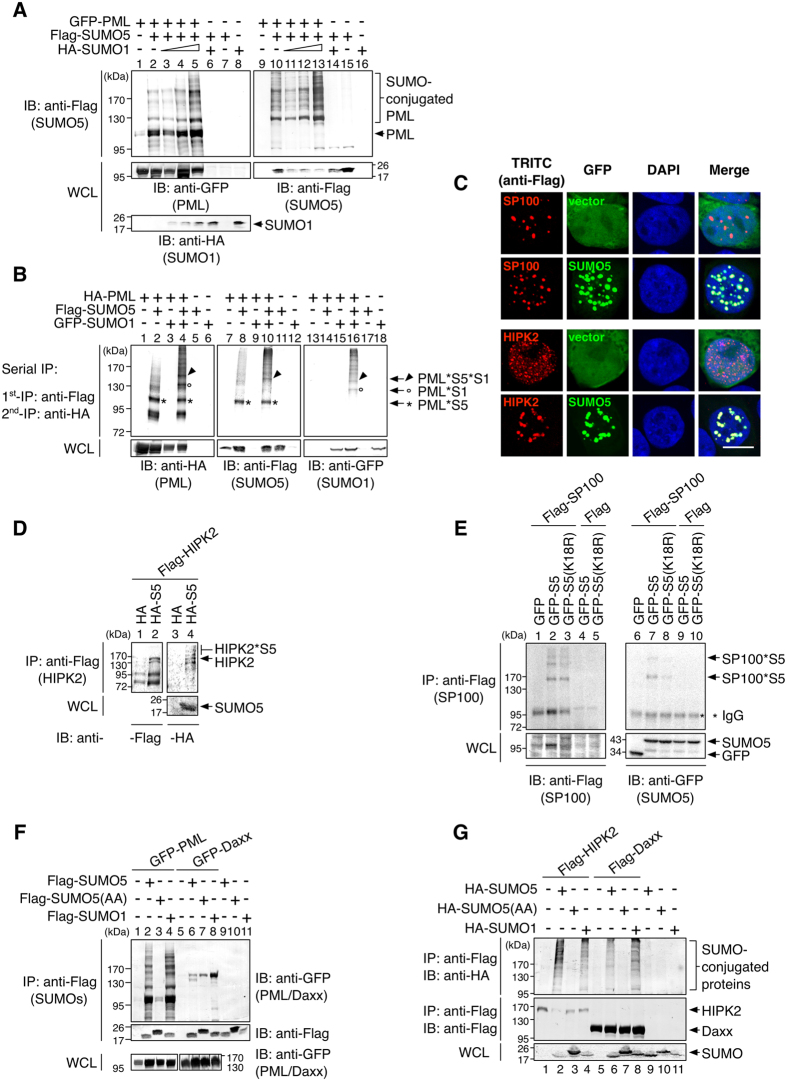
SUMO5 conjugation facilitates interaction among PML-NB components. (**A**) SUMO1 enhances SUMO5 conjugation of PML. 293 cells were transfected with GFP-PML, Flag-SUMO5, and increasing amounts of HA-SUMO1 expression plasmids. Anti-Flag immunoprecipitates were immunoblotted with anti-HA, anti-Flag, and anti-GFP antibodies as indicated. (**B**) SUMO1 and SUMO5 sequentially conjugate PML. 293 cells were transfected with HA-PML, Flag-SUMO5 and GFP-SUMO1 expression plasmids. Serial immunoprecipitation (serial IP) was performed using an anti-Flag antibody first and then anti-HA antibodies. Final immunoprecipitates were immunoblotted with anti-HA, anti-Flag, and anti-GFP antibodies as indicated. * : SUMO5-conjugated PML (PML*S5). ∙ : SUMO1-conjugated PML (PML*S1). ▴: SUMO5- and SUMO1-conjugated PML (PML*S5*S1). (**C**) SUMO5 enlarges nuclear domains of SP100 and HIPK2. 293 cells were transfected with Flag-tagged SP100 or HIPK2 together with GFP-SUMO5 or GFP vector. The formation of nuclear bodies containing SP100 or HIPK2 was assessed by confocal microscopy. Scale bar, 10 μm. (**D,E**) SUMO5 conjugates HIPK2 and SP100. Flag-tagged HIPK2 or SP100 were co-expressed with GFP-tagged SUMO5 or SUMO5(K18R). Anti-Flag immunoprecipitates were immunoblotted with anti-GFP or anti-Flag antibodies. HIPK2*S5 indicates SUMO5-conjugated HIPK2. SP100*S5 indicates different species of SP100 reflecting different degrees of SUMO5 conjugation. (**F**) SUMO5 interacts with PML and Daxx. Interaction of SUMO5 with PML or Daxx was analyzed by co-immunoprecipitation from lysates of 293 cells transfected with plasmids indicated. (**G**) Daxx and HIPK2 associate with SUMO5-conjugated proteins. 293 cells were transfected with expression plasmids indicated. SUMO-conjugated proteins associated with Daxx and HIPK2 were visualized by co-immunoprecipitation.

The major SUMO5 conjugation sites on PML (K160 and K490) overlapped the conjugation sites by SUMO1 reported in the literature. To further elucidate the differences between SUMO5 and SUMO1 regarding PML-NB formation, we performed SUMO conjugation assay and found that increasing amounts of SUMO1 enhanced conjugation of PML by SUMO5 but not vice versa (Figs 5A and S5A). Moreover, serial immunoprecipitation assay showed that both SUMO5 and SUMO1 could conjugate PML at the same time (Fig. 5B). These data suggest that conjugation of SUMO1 leads to increased conjugation of SUMO5 to PML.

To understand how increased SUMO5 conjugation to PML affected the formation of PML-NBs, we expressed SUMO5 and found that SUMO5 enlarged NBs containing SP100 and HIPK2 (Fig. 5C) as well as NBs containing SUMO1-3 (Fig. S5B). In contrast, expression of SUMO2/3 did not result in enlargement of PML-NBs (Fig. 3A). Co-immunoprecipitation assays showed that SUMO5 conjugated both HIPK2 (Fig. 5D) and SP100 (Fig. 5E). We confirmed that SUMO5 conjugated SP100 as monomers since SUMO5(K18R) mutant could conjugate SP100 (Fig. 5E, lanes 3 and 8). SUMO5 also interacted with PML and Daxx (Fig. 5F). Furthermore, HIPK2 and Daxx interacted with proteins conjugated with SUMO5 (Fig. 5G, lanes 2 and 6). These results indicate that SUMO5 promotes the enlargement of PML-NBs by recruiting, conjugating, and enhancing interaction among components of PML-NBs.

### SUMO5 conjugation of PML is replaced by SUMO2/3 during PML-NB disruption

**Figure 6:**
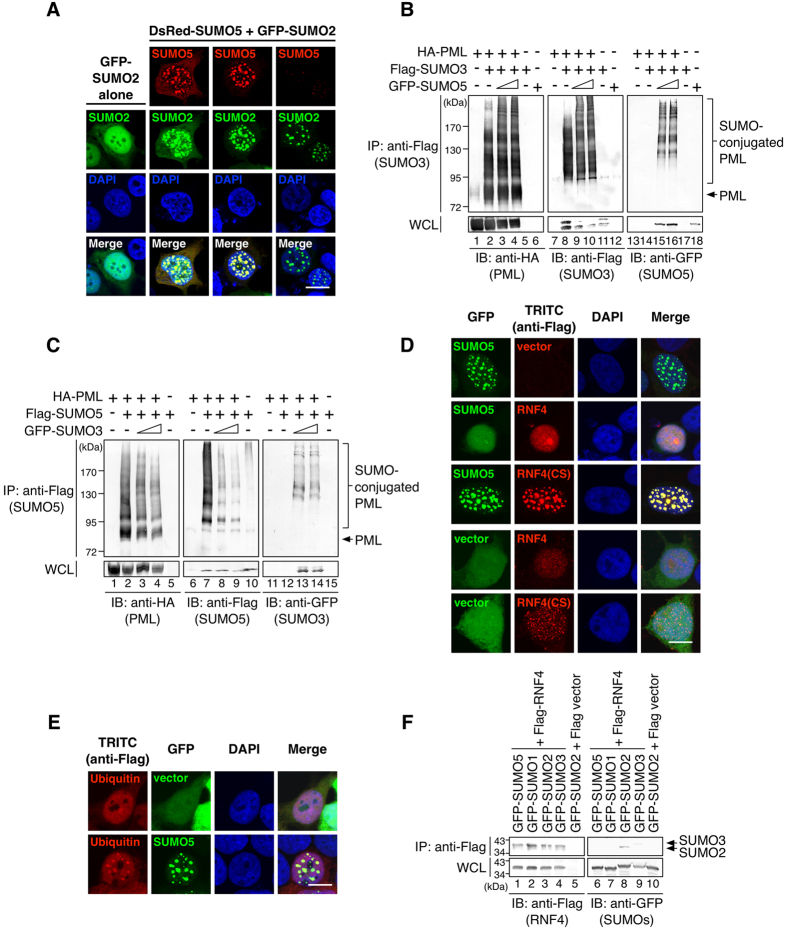
SUMO5 mediates the normal turnover of PML-NBs. (**A**) SUMO5 recruits SUMO2. GFP-tagged SUMO2 was transfected into 293 cells alone or with DsRed-tagged SUMO5. PML-NBs were assessed by confocal microscopy. (**B**) SUMO5 enhances poly-SUMO2/3 conjugation of PML. 293 cells were transfected with HA-PML, Flag-SUMO3, HA-Ubc9, and increasing amounts of GFP-SUMO5 expression plasmids. Immunoprecipitates with anti-Flag antibodies were immunoblotted with anti-HA, anti-Flag, and anti-GFP antibodies as indicated. (**C**) SUMO3 promotes deconjugation of SUMO5 from PML. 293 cells were transfected with HA-PML, Flag-SUMO5, and increasing amounts of GFP-SUMO3 expression plasmids. Cell lysates were analyzed as in (**B**). (**D**) SUMO5 induces disruption of PML-NBs in an RNF4-dependent manner. 293 cells were transfected with combinations of GFP-SUMO5, Flag-RNF4, Flag-RNF4(CS), or vector controls. Images were obtained by confocal microscopy. (**E**) Ubiquitin is recruited into SUMO5-induced PML-NBs. 293 cells expressing Flag-ubiquitin were cotransfected with GFP-SUMO5 or GFP vector alone. Cell images were obtained by confocal microscopy. (**F**) RNF4 interacts with SUMO2/3 only. Interaction of RNF4 with different SUMOs was analyzed by co-immunoprecipitation from cell lysates of 293 cells transfected with plasmids indicated. Scale bars, 10 μm.

Although SUMO5 facilities the growth of PML-NBs, there was heterogeneity among NBs co-expressing SUMO2/3 and SUMO5 (Fig. 6A). To differentiate the roles of SUMO5 and SUMO2/3, we used SUMO conjugation analysis and found that SUMO5 enhanced the conjugation of SUMO3 to PML (Fig. 6B), but SUMO3 conjugation promoted deconjugation of SUMO5 from PML (Fig. 6C). The polymeric SUMO chains on high-molecular-weight species of PML contained combinations of SUMO5 and SUMO3 (Fig. S6A, lane 13), reflecting a snapshot of PML-NBs of various sizes from an asynchronous cell population. It was shown that SUMO2/3 conjugation results in recruitment of the ubiquitin E3 ligase RNF4, polyubiquitination of PML and proteasome-dependent degradation of PML^15^,^22^. Therefore, we wanted to know if SUMO5 conjugation, which increased SUMO2/3 conjugation, would result in the degradation of PML-NBs. We found that RNF4 indeed mediated the disruption of SUMO5-induced PML-NBs, and the dominant negative mutant RNF4(CS) blocked the RNF4-mediated degradation of PML-NBs (Fig. 6D). Furthermore, SUMO5-induced PML-NBs recruited ubiquitin (Fig. 6E) and could be preserved by the proteasome inhibitor MG132 (Fig. S6B). Moreover, RNF4 preferentially interacted with SUMO2 but not SUMO5 (Fig. 6F). These results demonstrate that SUMO5 conjugation of PML increases SUMO2/3 conjugation, which leads to the recruitment of RNF4 and ubiquitin-dependent disintegration of PML-NBs.

### Oncoprotein PML-RARα causes cytoplasmic localization of PML by competing with PML for SUMO5 conjugation

**Figure 7:**
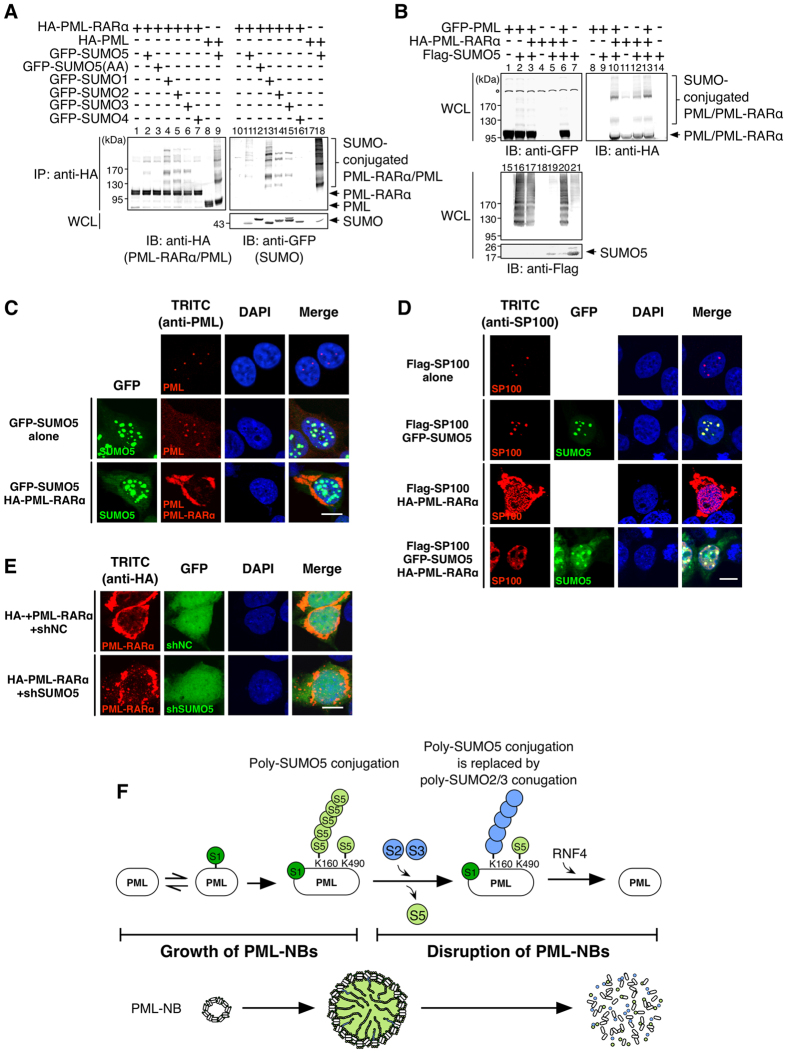
Competition for SUMO5 conjugation from PML-RARα causes cytoplasmic displacement of PML. (**A**) PML-RARα can be conjugated by SUMO5, SUMO1, SUMO2, SUMO3, but not by SUMO4. 293 cells were transfected with plasmids indicated. Anti-HA immunoprecipitates from cell lysates were immunoblotted with anti-HA or anti-GFP antibodies. (**B**) PML-RARα outcompetes PML for SUMO5 conjugation. 293 cells were transfected with expression plasmids indicated. Cell lysates were immunoblotted with anti-GFP, anti-HA, or anti-Flag antibodies. o, cross-reacting bands of anti-GFP antibody. (**C**) SUMO5-conjugated PML-RARα moves PML into cytoplasmic aggregates. 293 cells were transfected with GFP-SUMO5 alone (SUMO5 alone) or co-transfected with GFP-SUMO5 and HA-PML-RARα (SUMO5 and PML-RARα). An anti-PML antibody (anti-PML staining) was used in confocal microscopy to obtain cell images. (**D**) SP100 remains largely localized to PML-null SUMO5 NBs after PML is recruited into the cytoplasm by PML-RARα. 293 cells expressing Flag-SP100 were cotransfected with GFP-SUMO5 (Flag-SP100/GFP-SUMO5), HA-PML-RARα (Flag-SP100/HA-PML-RARα), or GFP-SUMO5 and HA-PML-RARα (Flag-SP100/GFP-SUMO5/HA-PML-RARα). (**E**) Without SUMO5, PML-RARα fails to form cytoplasmic aggregates. 293 cells were co-transfected with HA-PML-RARα and SUMO5 shRNA targeting endogenous SUMO5 or control shRNA (shNC). The shRNA constructs contained the GFP gene, and green fluorescence indicated expression of shRNAs. Localization of HA-PML-RARα was revealed by anti-HA staining in confocal microscopy. Scale bars, 10 μm. (**F**) PolySUMO5 conjugation regulates the formation and disruption of PML-NBs. Enlargement of PML-NBs is facilitated by polySUMO5 conjugation of PML, which recruits other proteins into PML-NBs. PolySUMO5 conjugation also promotes polySUMO2/3 conjugation, which replaces SUMO5 conjugation on PML. De-conjugation of SUMO5 and polySUMO2/3 conjugation then initiate RNF4-dependent disruption of PML-NBs.

We have shown that SUMO5 mediated the formation and normal turnover of PML-NBs (Figs 3, 4, 5, 6). Moreover, SUMO5 was highly expressed in peripheral blood lymphocytes and the APL cell line NB4 (Fig. 1). Therefore, we hypothesized that SUMO5 was involved in the disruption of PML-NBs in the presence of oncoprotein PML-RARα (Fig. S7A). We first confirmed that PML-RARα could be conjugated by SUMO5 (Fig. 7A). We also found that PML-RARα had a greater ability to conjugate SUMO5 than PML when they were co-expressed (Fig. 7B, compare lanes 3 and 13). When PML and PML-RARα were both present in a cell, PML was de-localized into cytoplasmic aggregates with PML-RARα (Figs 7C and S7B). Other SUMOs and classical components of PML-NBs such as SP100 were partially de-localized into the cytoplasm by PML-RARα (Figs 7D and S7C). However, expression of SUMO5 kept SP100 and HIPK2 in the PML-Null NBs (Figs 7D and S7D,E), which resembled the phenomenon that arsenite degraded PML and left SUMO at the core of the NBs^34^. Knocking down SUMO5 severely compromised the formation of PML-RARα cytoplasmic aggregates (Figs 7E and S7F). These results show that SUMO5 is required for the formation of PML-RARα aggregates and suggest that PML-RARα removes SUMO5 conjugation specifically from PML, causing cytoplasmic displacement of PML but sparing other major components of PML-NBs that are SUMO5-conjugated (Fig. S7G).

## Discussion

### PolySUMO5 conjugation at K160 of PML is critical for PML-NB formation

SUMO5 polyconjugates PML by successive self-conjugation at K18, and the internal SUMO modification motif (IKDED) of SUMO5 containing K18 is required for PML-NB formation (Figs 2 and 3). Furthermore, mass spectrometric and biochemical analyses show that polySUMO5 conjugation happens at K160 of PML (Fig. 4). Given that polySUMO5 conjugation leads to PML-NB formation, K160 should be the critical residue in PML that controls the formation of PML-NBs. This is in agreement with a previous finding that K160 is the key lysine for PML-NB formation^12^,^14^,^15^, and it also explains why PML(K160R), which retains SUMO1 conjugation at other lysine residues, is unable to recruit Daxx or SP100 to form NBs^16^,^17^. Based on our results, we propose that SUMO5 is a novel SUMO that facilitates the formation of PML-NBs in human cells (Figs 7F and S7H): As SUMO1 generally expresses more than SUMO5 (Fig. S1D) and all six lysines of PML can be conjugated by SUMO1, SUMO1 conjugation of PML might serve to nucleate the formation of PNL-NBs by facilitating dimerization of PML. PolySUMO5 conjugation, which replaces SUMO1 conjugation at K160 of PML, recruits NB component proteins and causes PML-NBs to grow. Using our model, the discrepancies between previous results that PML(3K) can be conjugated by (Fig. S4E) and colocalized with SUMO1 in NBs^19^ but cannot form mature PML-NBs^17^ can be successfully reconciled.

### De-conjugation of SUMO5 initiates the disruption of PML-NBs

Our results show that replacement of the SUMO5 conjugation of K160 on PML by SUMO2/3 mediates the disruption of PML-NBs. This transition from polySUMO5 conjugation to polySUMO2/3 conjugation recruits RNF4 to facilitate the disruption of PML-NBs. These data explain how PML-NBs disrupt under normal conditions, which resembles a previous PML-NB disruption model under oxidative stress^15^. Both models contain conjugation by SUMO2/3 and the involvement of RNF4. It is important to point out that under normal cell culture conditions, we saw no sign of PML degradation. This is consistent with previous reports that PML-NBs are disrupted by relocating and recycling PML during cell cycle progression^10^,^35^. According to these findings, it is likely that during normal cell cycling, RNF4 targets protein factors required for the integrity of PML-NBs rather than PML itself, and the disappearance of PML-NBs in our assays is through disruption of PML-NBs rather than degradation of PML. Further identification of these factors targeted by RNF4 might reveal the mechanism. Our findings here suggest that de-conjugation of SUMO5 from PML initiates the disruption cascade of PML-NBs through SUMO2/3-induced recruitment of RNF4 and polyubiquitination-dependent degradation of PML-NB components (Fig. S7H). However, signals trigger the de-conjugation of SUMO5 from PML requires further investigation.

### Differential conjugation of SUMO5 underlies the pathogenesis of APL

PML is conjugated by SUMO5 with a greater efficiency than PML-RARα when they are expressed individually (Fig. 7A). However, when PML and PML-RARα co-express, PML-RARα is more readily conjugated by SUMO5 than PML, resulting in a preferential loss of PML SUMOylation (Fig. 7B). Furthermore, PML is translocated to cytoplasmic aggregates with PML-RARα, suggesting that the removal of SUMO5 conjugation from PML by PML-RARα is linked to the cytoplasmic translocation of PML. Combining these results with a recent finding that PML-RARα disrupts the dimerization of PML^36^, we propose that in APL cells, PML-RARα interferes with the dimerization of PML and removes SUMO5 conjugation from PML, meanwhile taking PML to the cytoplasm with it. This process blocks both the nucleation of PML-NBs and the normal functions of PML in the nucleus. It also adds a new layer of regulation to the pathogenesis of APL on top of the abnormality in transcription caused by the absence of PML and the presence of PML-RARα in the nucleus. It is important to note that some SUMO5 still resides in the nucleus despite the expression of PML-RARα, and these SUMO5 molecules conjugate other targets to form NBs without PML. This is consistent with our hypothesis that SUMO5-conjugated protein factors other than PML are targeted by RNF4 to cause disruption of PML-NBs (see above). As a corollary, SUMO5-conjugated proteins besides PML might also regulate the biogenesis of PML-NBs, which requires further examination to confirm.

### Possible roles of SUMO5 in DNA damage response

Previous studies have reported that PML-NBs are stress-induced subnuclear structures^37^. As SUMO5 regulates the life cycle of PML-NBs and polySUMO5-conjugation of PML controls enlargement of PML-NBs (Fig. 2E), we reason that SUMO5 might be involved in biological processes that enlarge PML-NBs, including DNA damage response (DDR). Upon DNA damage, PML-NBs increase in both size and number^38^ and so do SUMO5-NBs (Fig. S8A,B), indicating that SUMO5 is stress-inducible. Interestingly, DNA damage-induced SUMO5 bodies do not colocalize with SUMO1 in nucleoli (Fig. S8A, bottom panel) or γ-H2AX foci (Fig. S8C), implying functional differences between SUMO1 and SUMO5 during DDR. SUMO1-conjugation has been well-characterized to play indispensable roles in DDR^39^, including modifications and activations of various DDR factors. However, less is known about how PML-NBs are induced during the process. SUMO5 does not seem to participate in the formation of repair foci (Fig. S8D). Rather, it affects recruitment of DDR factors to the repair foci (Fig. S8E). It is possible that the main function of SUMO5 in DDR is to orchestrate the formation of PML-NBs to serve as reserves for DDR factors while the function of SUMO1 is to modify these DDR factors. Further experiments are needed to confirm the role of SUMO5 in DNA damage response.

### Species- and tissue-specific expression of SUMO5

Although SUMO5 is highly homologous to SUMO1, it differs from SUMO1 in several remarkable aspects: First, it is conserved among primate species but appears not to be expressed in mice. Second, it is transcriptionally active in selective tissues, with high expression in testes and in blood cells. Theses attributes suggest that the mechanism regulating the life cycle of PML-NBs in human might differ from that in mice. However, despite persistent effort, we were unable to produce an antibody specific for SUMO5 due to the extremely high degree of identity between SUMO5 and SUMO1. Nevertheless, by using shRNA specifically targeting SUMO5, we showed SUMO5 was a critical regulator of the PML-NB cycle. Our study, which is the first comprehensive analysis of this novel member of the SUMO family, provides critical mechanistic details about the life cycle of PML-NBs.

### The expression of SUMO5 is under the regulation of multiple transcription factors

Through bioinformatic interrogation of the sequence upstream from the *SUMO5* gene, we found that the putative *SUMO5* promoter contains two C/EBP-β bindings sites and nine GATA-1 binding sites among other transcription factor binding motifs. This information suggests that *SUMO5* is potentially under the regulation of multiple transcription factors in normal, physiologic conditions, with C/EBP-β and GATA-1 being the most prominent regulators of SUMO5 expression. C/EBP-β is critical for the differentiation of adipocytes^40^ and macrophages^41^ while GATA-1 is most noted for its requirement for the maturation of erythrocytes^42^,^43^. Therefore, we suspect that proper regulation of the expression of SUMO5 is important in hematopoiesis and inflammatory responses.

Our analysis on how PML-RARα highjacks SUMO5 conjugation demonstrates that the control of SUMO5 conjugation is important in the pathogenesis of APL. Our data also show that SUMO5 is abundantly expressed in the APL cell line NB4 (Fig. 1C). Interestingly, the expression of C/EBP-β is induced in NB4 cells when they are treated with the cancer therapy drug ATAR^44^. We suspect that in pathological conditions like APL, the expression of *SUMO5* is controlled by transcription factors including C/EBP-β, whose own expression might also change when normal cell physiology is perturbed. As a result, the abnormal pattern of SUMO5 conjugation on target proteins such as PML and PML-RARα will likely to be reinforced in APL.

## Methods

### Cloning of SUMO5

SUMO5 cDNA was identified by RT-PCR using primers based on the coding region of SUMO1 cDNA and a cDNA library prepared from total RNA of 293 cells. Subsequently, PCR products were cloned in-frame into a GFP fusion expression vector (pEGFP-C1). Each clone was transfected into 293 cells. SUMO5 (GenBank accession number: FJ042790.1) was specifically selected based on the formation of NBs.

### Plasmid constructs

Coding regions of SUMO5, SUMO1, SUMO2, SUMO3, and SUMO4 were amplified from 293 cells by RT-PCR and then inserted into pEGFP-C1 (Clontech) to create plasmids expressing GFP-tagged SUMO proteins. cDNA fragments of SUMO5, SUMO1, and SUMO3 were subcloned from pEGFP-C1 into pDsRed1-C1 (Clontech), pmCherry-C (Clontech), pcDNA3.1-HA, and pcDNA3-Flag vectors to obtain plasmids expressing RFP-, mCherry-, HA-, and Flag-tagged proteins. The plasmid expressing GFP-tagged SUMO5(AA) was prepared using site-directed mutagenesis (QuikChange, Agilent) and the fragment of SUMO5(AA) was further subcloned into pcDNA3.1-HA and pcDNA3-Flag vectors. The coding region of ubiquitin was amplified from 293 cells by RT-PCR and then inserted into pcDNA3-Flag to create the plasmid expressing Flag-tagged ubiquitin. GFP-tagged Daxx, HA-tagged PML-RARα, and cDNAs of PML (GenBank accession number: M73778.1) and PML(3K) were kindly provided by Dr. Hsiu-Ming Shih. Flag-PML-I, Flag-PML-IV, and Flag-PML-VI were kindly provided by Dr. Ruey-Hwa Chen. Plasmids expressing Flag-, HA-, GFP-tagged PML and PML(3K) proteins and plasmids expressing Flag- or HA-tagged Daxx proteins were obtained by subcloning into pcDNA3-Flag, pcDNA3.1-HA, and pEGFP-C1 vectors. Plasmids expressing Flag-tagged SP100 and HIPK2 were provided by Dr. M. Lienhard Schmitz. Flag-tagged SENP1 was kindly provided by Dr. Kung-Yao Chang. Plasmids expressing HA-tagged Ubc9, PIAS1, and PIASx-α were kindly provided by Dr. Keith D. Robertson. Site-directed mutagenesis was performed to obtain the following mutants: SUMO5(H12D), SUMO5(K18R), SUMO5(I95T), SUMO5(sm), SUMO1(IKDE), SUMO1(IKDΔ), SUMO5(I95R), PML(K65R), PML(K160R), PML(K380R), PML(K400R), PML(K490R), PML(K497R), and Ubc9(DN)(C93S). pGEX-2TK-hPc2, the plasmid expressing GST-tagged Pc2, was kindly provided by Dr. David P. E. Satijn. HA-tagged Pc2 was obtained by insertion of a PCR-amplified DNA fragment of Pc2 into pcDNA3.1-HA vector. Plasmids expressing Flag-tagged RNF4 and its mutant RNF4(3CS) were kind gifts of Dr. Ronald T. Hay.

### Complete nucleotide sequences of primers for RT- PCR and genomic PCR

SUMO5

Forward: GGGATAAGATAAAAGATGAAG

Reverse: GTTGAATGACCCCCCGATTTG

SUMO1

Forward: CGGGATCCATGTCTGACCAGG

Reverse: GCTCTAGACTAAACTGTTGAATGAGCCGCCGTTTG

GAPDH (in Figs 1D, S1B, and S1C)

Forward: TGAAGGTCGGAGTCAACGGATTTGGT

Reverse: CATGTGGGCCATGAGGTCCACCAC

GAPDH (in Fig. 1C)

Forward: GGGTCATCATCTCTGCCCCC

Reverse: GGATGACCTTGCCCACAGCC

### Cell culture, transfection and treatments

The following cell lines were grown in DMEM with 10% fetal bovine serum and 1% penicillin-streptomycin: 293 (human embryonic kidney), HeLa (human cervix adenocarcinoma), MCF7 (human breast adenocarcinoma), A2058 (human metastatic melanoma), K562 (human chronic myelogenous leukemia), NIH 3T3 (mouse embryonic fibroblast), and B16F10 (mouse skin melanoma) cells. NB4 (human acute promyelocytic leukemia) cells were grown in RPMI with 20% fetal bovine serum and 1% penicillin-streptomycin. Expression plasmids were transfected into 293 cells using a calcium phosphate co-precipitation method. N-ethylmaleimide (NEM, Sigma, E3876), dissolved in 95% ethanol and stored at a concentration of 0.4 M at −20 °C, was used at a concentration of 10 mM by adding into the cell lysis buffer.

### Gene expression analysis

For the analysis of SUMO5 expression in cell lines, total RNA was isolated from 293, K562, NB4, A2058, MCF7, HeLa, B16F10, and NIH 3T3 cells using a WelPrep Cell/Tissue RNA Kit (Welgene). Extracted RNAs were used as template in reverse transcriptase PCR with oligo-dT primers and NxGen™ M-MuLV Reverse Transcriptase (Lucigen), and followed by PCR with the SUMO5-specific primer pair. For the analysis of SUMO5 expression in tissues, cDNAs from Multiple Tissue cDNA Panels Human I and II (Clontech) were used as template for PCR. GAPDH was used as internal control in Figs 1C–E and S1B,C. To detect SUMO5 expression, the same parameters of touchdown PCR were used in all expression profiling procedures: 94 °C 2′ start-up, followed by 3 cycles of 94 °C 30″/55 °C 30″/72 °C 30″, followed by 3 cycles of 94 °C 30″/53 °C 30″/72 °C 30″, followed by 3 cycles of 94 °C 30″/51 °C 30″/72 °C 30″, followed by 3 cycles of 94 °C 30″/49 °C 30″/72 °C 30″, followed by 25 cycles of 94 °C 30″/47 °C 30″/72 °C 30″, followed by 72 °C 5′, and finally stored at 4 °C.

### shRNA knockdown

Small hairpin RNA (shRNA) constructs shSUMO1/5 (RHS4430-98481086), shUbc9 (RHS4430-98520611), shSUMO5 (customized SUMO5-specific shRNA) and shNC (negative control shRNA) were obtained from GenDiscovery Biotechnology. The target sequences of shSUMO1/5, shUbc9, shSUMO5, and shNC are TTATCAGCAATTCTCTGAC, TTAGTGAAGCCACAGATGTAA, GGGATAAGATAAAAGATGAAG, and TTCTCCGAACGTGTCACGT, respectively. shRNA was delivered into cells by calcium phosphate co-precipitation. In the rescue assay, shSUMO1/5 was first transfected into 293 cells. 24 hours later, the culture media were discarded and cells were washed three times with PBS prior to the second transfection with the SUMO5 or SUMO1 expression construct.

### Antibodies

Mouse monoclonal anti-HA (H9658) and anti-FLAG M2 (F1804) antibodies were from Sigma-Aldrich. Mouse monoclonal anti-SUMO1 (sc-5308), mouse monoclonal anti-hPML (sc-966), goat polyclonal anti-hSP100 (sc-16328), mouse monoclonal anti-gamma-tubulin (sc-17788), mouse anti-β-actin (sc-47778), and rabbit polyclonal anti-hRAR-α (sc-551) antibodies were from Santa Cruz Biotechnology. Mouse anti-KAP1 antibody was made in house. Rabbit polyclonal anti-GFP antibody was a kind gift from Dr. Liang-Jwu Chen. Mouse anti-GFP monoclonal antibody clone 3D8A1B8 (Gm0001-02) was from Abking Biotechnologies Inc.

### Serial immunoprecipitation

To perform serial IP, anti-Flag IP was carried out first using standard procedures. After washing, immunoprecipitated proteins were eluted by adding 300 μl of PBS containing 0.1% NP-40 and 100 μg/ml of a Flag peptide (Sigma, F3290) for 30 min on ice. After pulse centrifugation (5 sec at 12,000 rpm), the soluble phase was collected for anti-HA IP.

### Indirect immunofluorescence and confocal laser microscopy

293 cells were grown on glass coverslips in 6 cm dishes and transfected as described. After 24 hours, cells were treated with reagents for the time indicated and fixed with 3% paraformaldehyde for 10 min at room temperature at indicated time points. Fixed cells were washed with PBS once and permeabilized with 0.2% Triton X-100 in PBS. For immunolocalization of endogenous proteins, cells were incubated with primary antibodies and fluorescently labeled secondary antibodies. Fluorescence microscopy analysis was carried out using an Olympus A BX51 upright epi-fluorescence microscope with an UplanFL 100× objective. Confocal microscopy analysis was carried out with an Olympus FluoView FV1000 laser-scanning microscope using a 63 × 1.4 numerical aperture objective.

### Protein complex purification

Approximately 1.7 × 10^8^ 293 cells expressing Flag-SUMO5 were lysed in PBS containing 0.1% NP-40 followed by centrifugation at 12,000 rpm for 10 min to obtain a cleared lysate. The lysate was applied to a gravity column (Bio-Rad Laboratories) containing 600 μl of anti-Flag M2-Agarose (Sigma, A2220) and re-applied for a total of three times. The affinity column was washed extensively with the lysis buffer, and the bound SUMO5 complex was eluted from the column with 3 ml of an elution buffer containing 20 μg/ml of Flag peptide (Sigma, F3290). Five fractions of eluate were obtained, and the third fraction was air-dried to concentrate to the proper volume before SDS-PAGE separation using 3% stacking/12% resolving polyacrylamide gels. Protein bands were stained with silver for visualization.

### Mass spectrometric analysis

In-gel digestion of selected protein bands was performed essentially as described^45^. Digested peptides were introduced into a ThermoFisher Scientific LTQ XL (San Jose, CA) linear ion trap mass spectrometer equipped with a nanoelectrospray ion source via high-performance liquid chromatography using an Agilent (Palo Alto, CA) 1200 series binary HPLC pump and an LC packings FAMOS^TM^ well-plate microautosampler. A full-mass scan was performed between m/z 350 and 2000, followed by MS/MS scans of the five highest-intensity parent ions at 35% relative collision energy. The AGC of fluoranthene anions was set at 3 × 10^5^ and the reaction time was set at 200 ms during ETD.

For the identification of proteins in the SUMO5 complex, the acquired MS/MS spectra were searched against the Swiss-Prot protein database (released 8 January 2008) using Mascot Daemon version 2.2.2. *Homo sapiens* (human, 17,869 sequences) was chosen as the taxonomic category. For the identification of SUMO5 modification sites on PML, the acquired MS/MS spectra were searched against Swiss-Prot (released 27 August 2013) using Mascot Daemon version 2.3.2. *Mammalia* (Mammalia, 66,299 sequences) was chosen as the taxonomic category. Masses for both precursor and fragment ions were treated as monoisotopic. In addition, an in-house database containing SUMO5 (GI: 52783791), SUMO1 (GI: 52783799), PML-IV (NCBI name: PML isoform 6; GI: 4505903), and Pc2 (GI: 55770830) was used.

## Additional Information

**Accession codes**: The *SUMO5* gene (GenBank: FJ042790.1); *SUMO1* gene (GenBank: NG_011679.1); SUMO1 protein (GenBank: AAC50996.1); SUMO2 protein (GenBank: AAH71645.1); SUMO3 protein (NCBI Reference Sequence: NP_008867.2); SUMO4 protein (NCBI Reference Sequence: NP_001002255.1); Homo sapiens promyelocytic leukemia (PML), transcript variant 6 (alternative name PML-IV, NCBI Reference Sequence: NM_002675.3). In Fig. 1B, the sequence accession numbers of SUMO5 orthologs are: Human (*Homo sapiens*, GenBank accession ID: GCA_000001305.2), Chimp (*Pan troglodytes*, GenBank accession ID: GCA_000001515.4), Bonobo (*Pan paniscus*, UCSC version: Max-Planck/panPan1, ftp://hgdownload.cse.ucsc.edu/gbdb/panPan1), Gorilla (*Gorilla gorilla gorilla*, GenBank accession ID: GCA_000151905.1), Orangutan (*Pongo pygmaeus abelii*, GenBank accession ID: GCF_000001545.3), Gibbon (*Nomascus leucogenys*, GenBank accession ID: GCA_000146795.2), Rhesus (*Macaca mulatta*, GenBank accession ID: GCA_000230795.1), Crab-eating macaque (*Macaca fascicularis*, UCSC version: WashU 5.0/macFas5, ftp://hgdownload.cse.ucsc.edu/gbdb/macFas5), Baboon (*Papio anubis*, GenBank accession ID: GCA_000264685.1), Green monkey (*Chlorocebus sabaeus*, UCSC version: VGC Chlorocebus_sabeus 1.1/chlSab2, ftp://hgdownload.cse.ucsc.edu/gbdb/chlSab2), Proboscis monkey (*Nasalis larvatus*, UCSC version: PMFGC Charlie1.0/nasLar1, ftp://hgdownload.cse.ucsc.edu/gbdb/nasLar1), Golden snub-nosed monkey (*Rhinopithecus roxellana*, UCSC version: Rrox_v1/rhiRox1, ftp://hgdownload.cse.ucsc.edu/gbdb/rhiRox1), Marmoset (*Callithrix jacchus*, GCA_000004665.1), Squirrel monkey (*Saimiri boliviensis*, GenBank accession ID: GCA_000235385.1), Tarsier (*Tarsius syrichta*, GenBank accession ID: GCA_000164805.2), Mouse lemur (*Microcebus murinus*, GenBank accession ID: GCA_000165445.1), Bushbaby (*Otolemur garnettii*, GenBank accession ID: GCA_000181295.3), Tree shrew (*Tupaia belangeri*, GenBank accession ID: GCA_000181375.1), Mouse (*Mus musculus*, GCA_000001635.2), Dog (*Canis lupis familiaris*, GenBank accession ID: GCA_000002285.2).

**How to cite this article**: Liang, Y.-C. *et al.* SUMO5, a Novel Poly-SUMO Isoform, Regulates PML Nuclear Bodies. *Sci. Rep.* **6**, 26509; doi: 10.1038/srep26509 (2016).

## Supplementary Material

Supplementary Information
