# Supplementary material for: SUMO5, a Novel Poly-SUMO Isoform, Regulates PML Nuclear Bodies
Source: Sci Rep. 2016 May 23;6:26509. doi: 10.1038/srep26509 (PMC4876461; doi:10.1038/srep26509)
Supplement: Supplementary Information [file srep26509-s1.pdf]

SUMO5, a Novel Poly-SUMO Isoform, Regulates PML Nuclear Bodies

Ya-Chen Liang[1], Chia-Chin Lee[1], Ya-Li Yao[2,*], Chien-Chen Lai[1], M. Lienhard Schmitz[3], and Wen-Ming Yang[1,*]

[1]Institute of Molecular Biology, National Chung Hsing University, Taichung 40227, Taiwan

[2]Department of Biotechnology, Asia University, Taichung 41354, Taiwan,

[3]Institute of Biochemistry, Medical Faculty, Friedrichstrasse 24, Justus-Liebig-University, 35392 Giessen, Germany.

[*]Correspondence: yyao@asia.edu.tw (Y.-L. Y) and yangwm@nchu.edu.tw (W.-M. Y)

Supplementary Information

A

```
ATG TCT GAC CTG GAG GCA AAA CCT TCA ACT GAG CAT TTG GGG GAT AAG ATA AAA GAT GAA  < 60
M   S   D   L   E   A   K   P   S   T   E   H   L   G   D   K   I   K   D   E
GAT ATT AAA CTC AGG GTT ATT GGA CAG GAT AGC AGT GAG ATT CAT TTC AAA GTG AAA ATG  < 120
D   I   K   L   R   V   I   G   Q   D   S   S   E   I   H   F   K   V   K   M
ACA ACA CCT CTC AAG AAA CTC AAG AAA TCG TAC TGT CAG AGA CAG GGC GTT CCA GTG AAT  < 180
T   T   P   L   K   K   L   K   K   S   Y   C   Q   R   Q   G   V   P   V   N
TCC CTC AGG TTT CTC TTT GAA GGT CAG AGA ATT GCT GAT AAT CAT ACT CCA GAA GAA CTG  < 240
S   L   R   F   L   F   E   G   Q   R   I   A   D   N   H   T   P   E   E   L
GGA ATG GAG GAA GAA GAT GTG ATT GAG GTT TAT CAG GAA CAA ATC GGA GGT CAT TCA ACA  < 300
G   M   E   E   E   D   V   I   E   V   Y   Q   E   Q   I   G   G   H   S   T
GTT TAG
V   *
ACATTCTTTTTTTTTTTCCTTTTCCCTCAATCCTTTTTTATTTTTTTAAATAGTTCTTTTGTAGCATGGTATTTAAAACGGAA
TTGAAAACAGGCACTCCATCTCTTTAAAACATCTGGTAATTTGAATTCTAGTGTTCATTATTCATTATTGTTTGTTTTCATTA
TGCTGATTTCTGGTGATCAAGGCTCAGGCCTCTTCATATTGCCCTCTCCTTTTATAAAAATTATGTGTGCACAGAGAGGCCAC
CTTTTTCAGGACTGTACATTTTCAGATGATAAGATTGACCAATGCAAGTATTCATAATGACTTTCCAGTTGGCCCTGATTTTC
AACCATGTGATTGTTTCACTCCTGGACTATGACTTTTGGTGGGAGATGGAAGTTTTTTAGAGAACTGAGCTGTGGAAAAATGA
CCTCTCCTTAACAACTTGAAGCTATTTTTAAGATTTGAGGGTCTGGACCAAAAGAAAAGGAGCATCAGGTTAAGTCAAGGTGA
CAGATAAGGTTGAGAGTAACGACTAACTCCAAAGATGGCTTCCCTGAAGAAAAGACATTTGAAGATTTTTTTTTTCTTAATCT
TGTCAGAAGATCCCAGAAAAGTTCTGATTTTCATTATCAATAAAGGTATACATGCAGAAATGAATACAACAGAATACTGCTCT
TTTTTATTTGTACTTTTTGGCCT
```

B **C** **D**

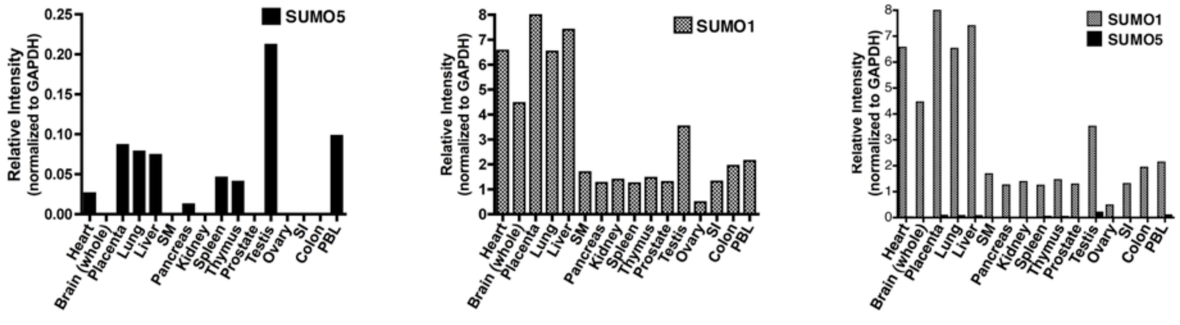

Figure S1. Expression of human SUMO5 is tissue-specific. Related to Figure 1.

(A) The nucleotide and predicted amino acid sequences of the human SUMO5 gene. The polyadenylation signal AATAAA is in bold and underlined. (B) Expression of SUMO5 is tissue-specific. Intensity of SUMO5 expression taken from the tissue blot (Fig. 1E) was normalized against that of GAPDH. (C) Expression of SUMO1 is not tissue-specific. Expression intensity of SUMO1 was analyzed as in (B). (D) Expression of SUMO5 is relatively low compared to expression of SUMO1 in tissues.

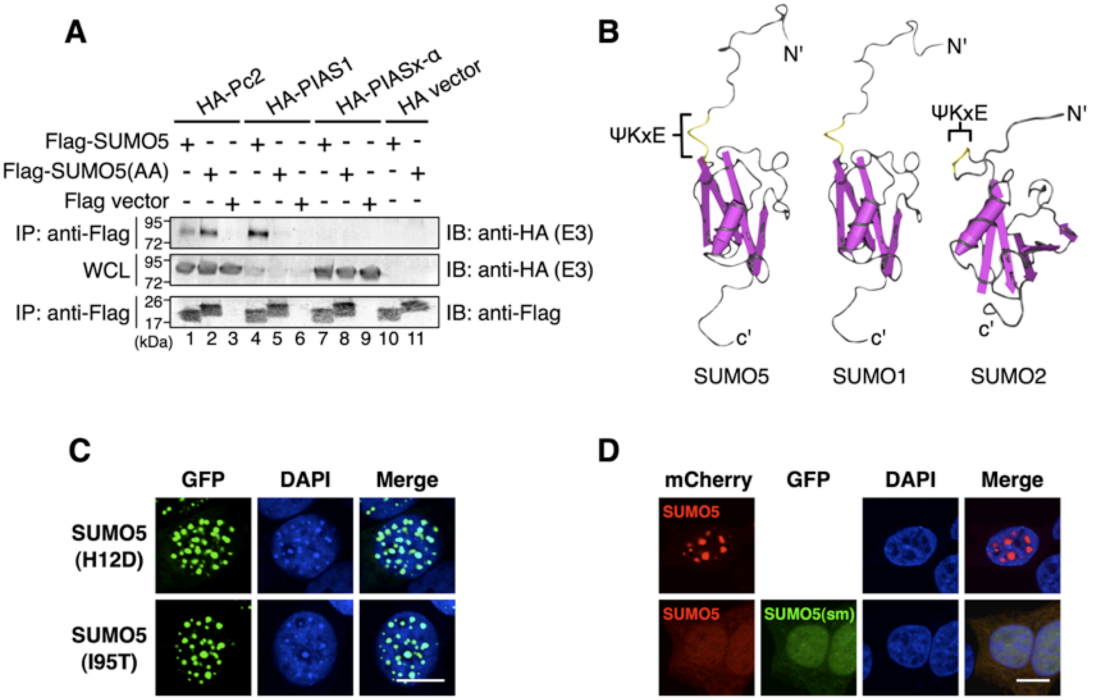

Figure S2. The conserved SUMO modification motif is required for SUMO5-mediated NB formation. Related to Figure 2.

(A) SUMO5 interacts specifically with SUMO E3 enzymes PIAS1 and Pc2. Flag-SUMO5 or Flag-SUMO5(AA) were cotransfected into 293 cells with HA-tagged Pc2, PIAS1, or PIASx-α. Anti-Flag immunoprecipitates (IP) of cell lysates were immunoblotted (IB) with anti-Flag or anti-HA antibodies as indicated. (B) Comparison of the protein structures of SUMO5, SUMO1, and SUMO2. The structures of SUMO1 (Protein Data Bank code 1A5R), SUMO2 (PDB code 2AWT), and SUMO5 (predicted structure using PDB code 1A5R as template) were generated using the Cn3D program. Magenta, secondary structures. Yellow, the conserved SUMO modification motif (ΨKXE). (C) SUMO5(H12D) and SUMO5(I95T) form nuclear bodies. Localization of GFP-tagged SUMO5(H12D) and SUMO5(I95T) and their associated NBs were assessed by confocal microscopy after transfection in 293 cells. DNA (blue) was stained with DAPI. (D) Co-expression of SUMO5 and SUMO5(sm) results in a loss of NBs. Scale bars, 10 µm.

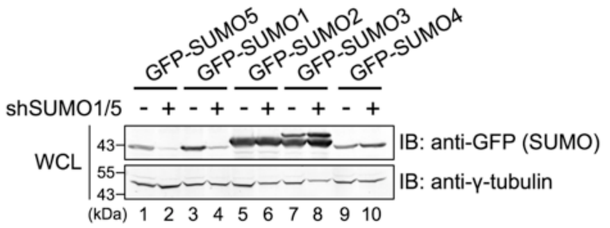

Figure S3. shSUMO1/5 knocks down expression of both SUMO5 and SUMO1. Related to Figure 3.

GFP-SUMOs were cotransfected into 293 cells with an shRNA construct targeting both SUMO1 and SUMO5 (shSUMO1/5). Whole cell lysates were immunoblotted with anti-GFP or anti-gamma-tubulin antibodies as indicated.

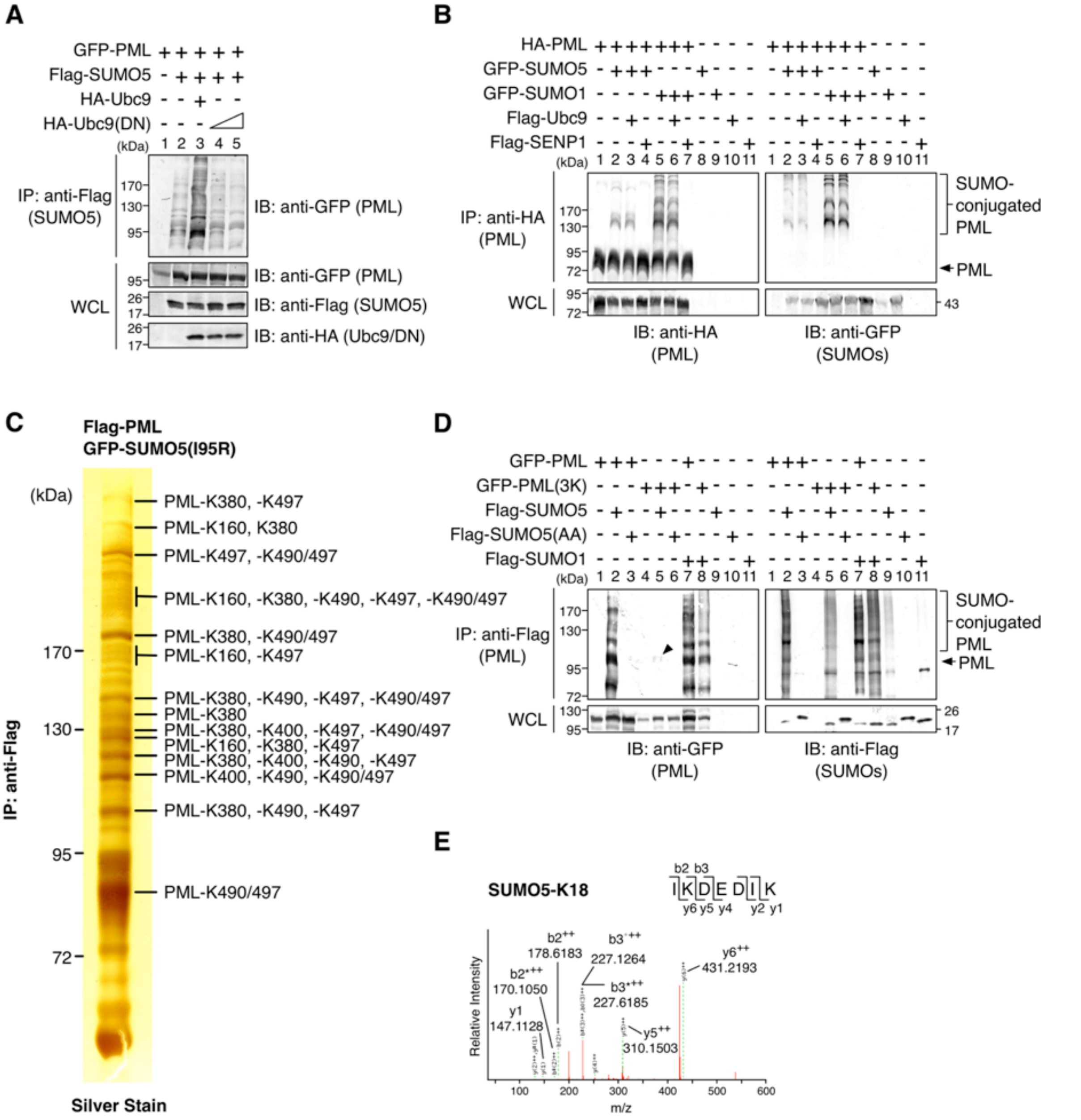

Figure S4. SUMO5 polySUMOylates PML. Related to Figure 4.

(A) Ubc9(DN) blocks SUMO5 conjugation of PML. GFP-PML, Flag-SUMO5, HA-Ubc9, and increasing amounts of HA-Ubc9(DN) expression plasmids were transfected into 293 cells. Anti-Flag immunoprecipitates were immunoblotted with anti-Flag, anti-HA, or anti-GFP antibodies as indicated. (B) The SUMO-specific protease SENP1 deconjugates SUMO5 from PML. 293 cells were transfected with combinations of expression plasmids as indicated. Anti-Flag immunoprecipitates of cell lysates were immunoblotted with anti-HA or anti-GFP antibodies. Protein bands corresponding to SUMOylated PML were marked as SUMO-conjugated PML. (C) K160, K380, K400, K490, and K497 of PML are SUMO5-conjugation sites on PML. Anti-Flag immunoprecipitates from 293 cell lysates expressing Flag-PML and GFP-SUMO5(I95R) were analyzed by 6% SDS-PAGE and silver staining. Protein bands specific to SUMO5(I95R)-conjugated PML were identified by LC-MS/MS. (D) PML(3K) can be conjugated by SUMO1. 293 cells were transfected with combinations of expression plasmids as indicated. Anti-HA immnunoprecipitates of cell lysates were immunoblotted with anti-Flag or anti-GFP antibodies. (E) SUMO5 self-conjugates at K18. Anti-Flag immunoprecipitates from 293 cell lysates expressing Flag-SUMO5(I95R) were subjected to tryptic digestion and LC-MS/MS analysis.
b2++ (m/z 178.6) and b2*++ (m/z 170.1), two fragment ions containing an internal lysine (K18), had a di-glycine diagnostic peptide. The mass difference (242.1 Da) between y6++ (m/z 431.2) and y5++ (m/z 310.2) also represents a di-glycine peptide at K18, further indicating conjugation of one SUMO5 molecule at K18 of another SUMO5 molecule.

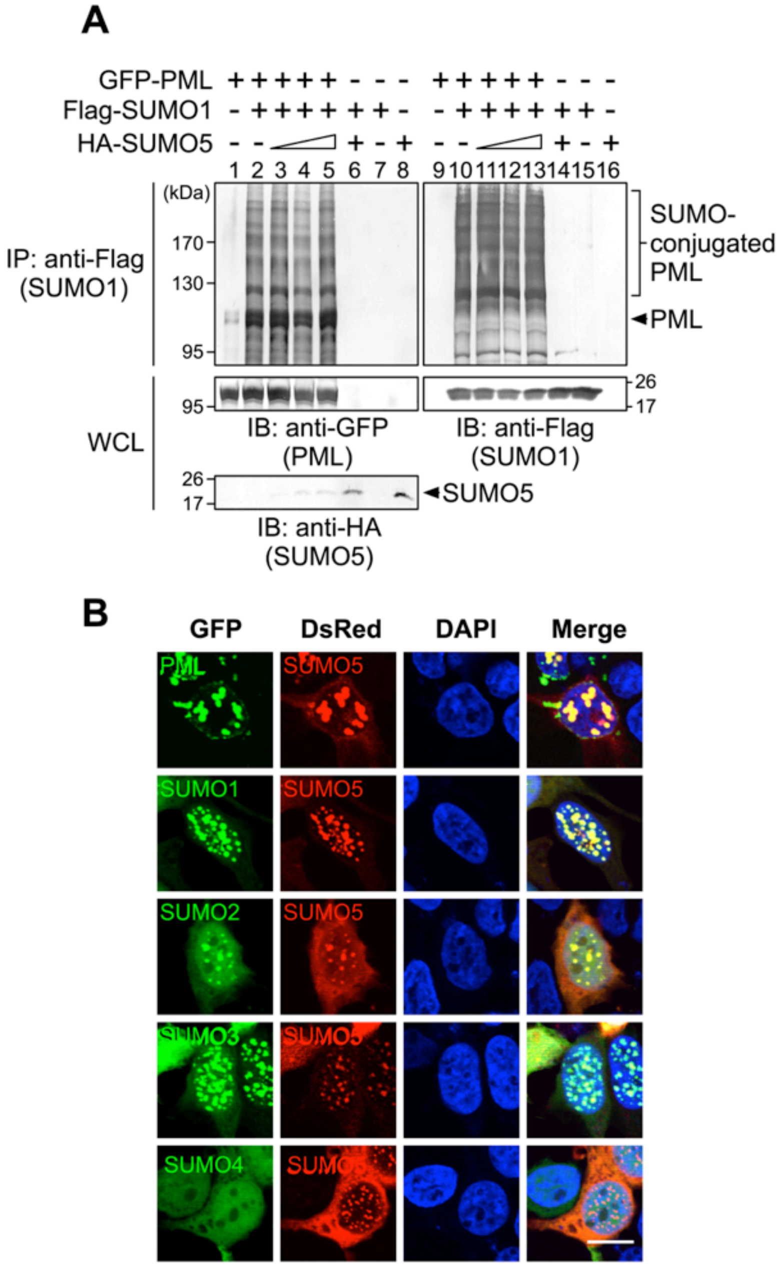

Figure S5. Multiple SUMO conjugations exist in SUMO5-induced PML-NBs. Related to Figure 5.

(A) SUMO5 does not affect SUMO1 conjugation of PML. 293 cells were transfected with GFP-PML, Flag-SUMO1, and increasing amounts of HA-SUMO5 expression plasmids. Immunoprecipitates with anti-Flag antibodies were immunoblotted with anti-HA, anti-Flag, and anti-GFP antibodies as indicated. (B) SUMO5 induced PML-NBs recruit SUMO1/2/3. RFP-tagged SUMO5 was cotransfected into 293 cells with GFP-tagged PML, SUMO1, SUMO2, SUMO3, or SUMO4. Recruitment by SUMO5-NBs was assessed by co-localization of red and green fluorescence in confocal microscopy.

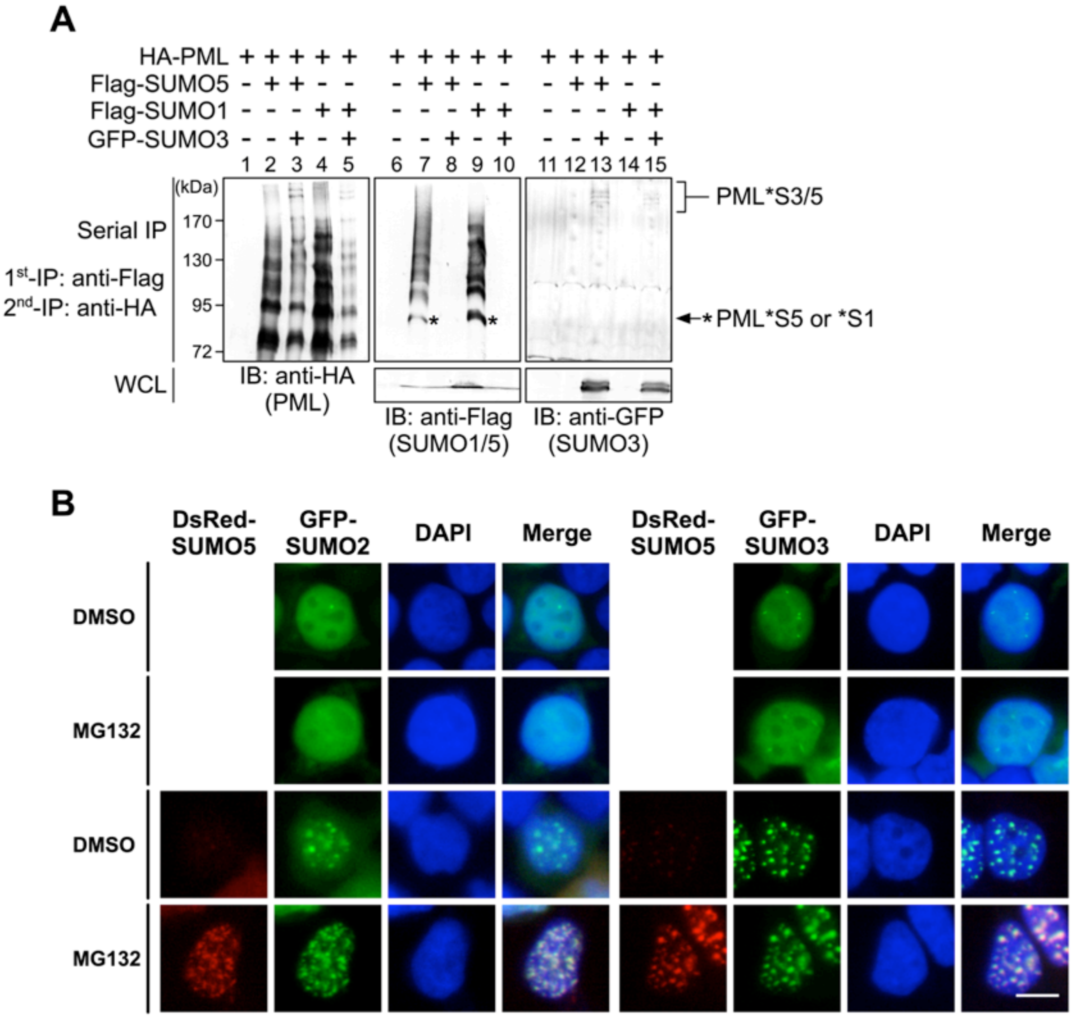

Figure S6. The normal turnover of PML-NBs is associated with the formation of polymeric SUMO5/3 chain on PML. Related to Figure 6.

(A) Coexpression of SUMO3 and SUMO5 causes the formation of polymeric SUMO5/3 chains on PML. 293 cells were transfected with HA-PML, Flag-SUMO5, Flag-SUMO1, and GFP-SUMO3 expression plasmids. Serial IP was performed using an anti-Flag antibody first and then an anti-HA antibody. Final immunoprecipitates were immunoblotted with anti-HA, anti-Flag, and anti-GFP antibodies as indicated. (B) MG132 blocks RNF4-mediated disruption of PML-NBs and prolongs the existence of SUMO5-mediated PML-NBs. 293 cells were transfected with either GFP-SUMO2/3 alone or co-transfected with DsRed-SUMO5. Transfected cells were treated with 5 μM of MG132 or solvent (DMSO) for 16 hours. Cells were then fixed and analyzed by immunofluorescence microscopy. Scale bars, 10 μm.

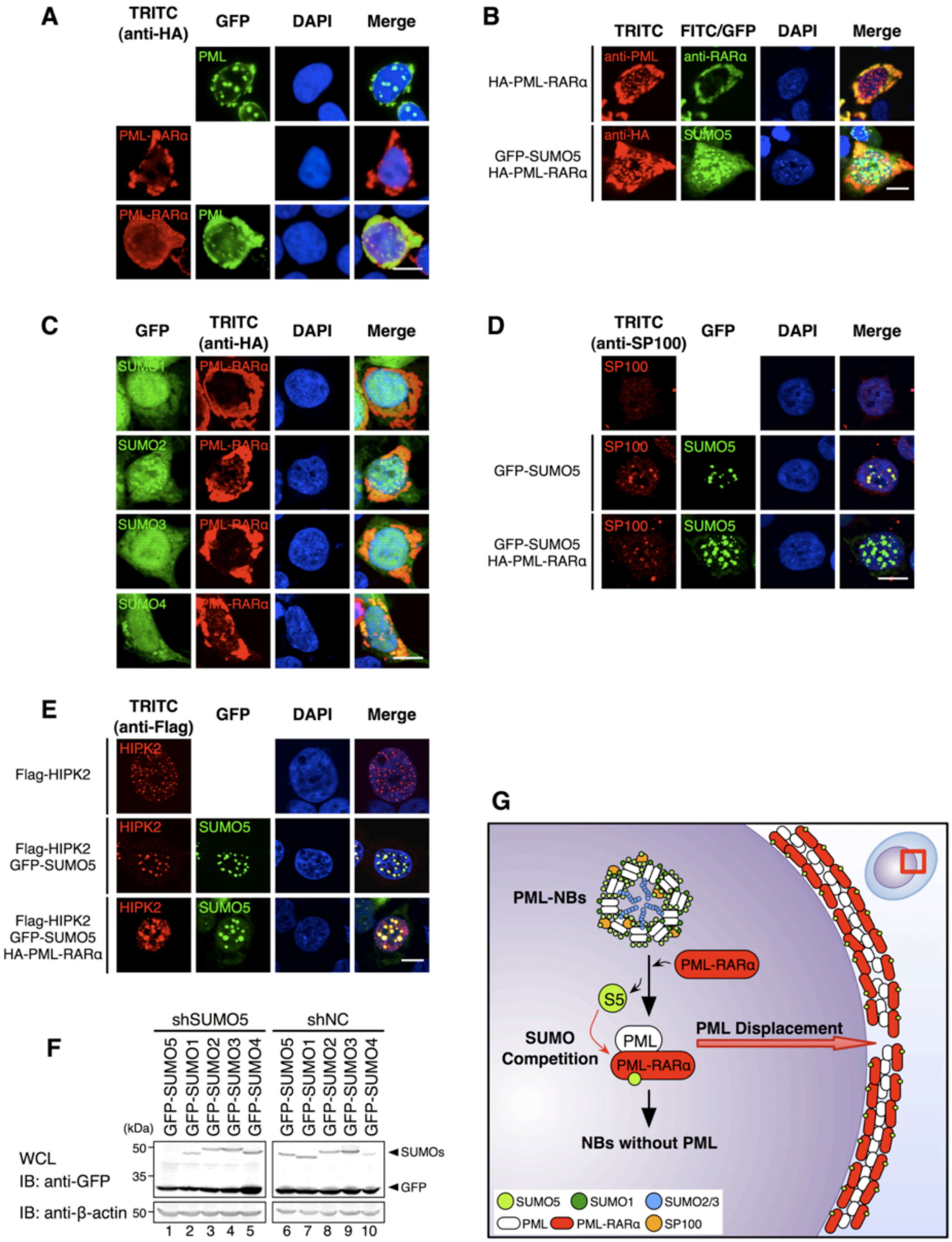

Figure S7. (This figure continues on the next page.)

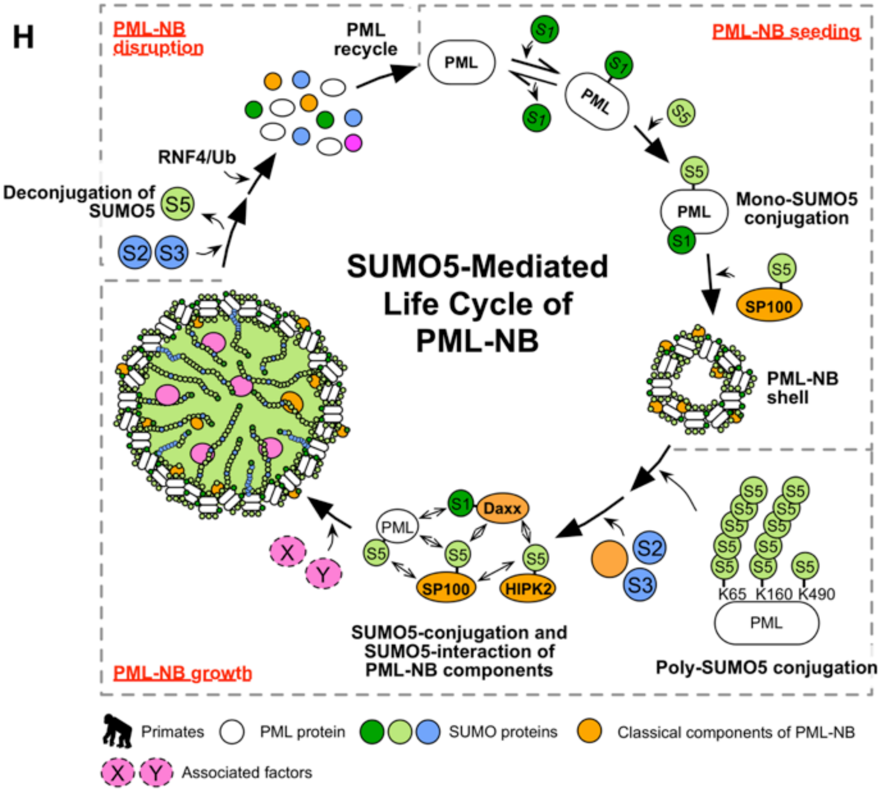

Figure S7. SUMO5-conjugated PML-RARα causes cytoplasmic displacement of PML and the formation of PML-null NBs. Related to Figure 7.

(A) PML-RARα disrupts the formation of PML-NBs. Subcellular localization of GFP-PML and HA-PML-RARα in 293 cells was analyzed by confocal microscopy. (B) PML-RARα moves PML and SUMO5 into cytoplasmic aggregates. Subcellular localization of endogenous PML (anti-PML), HA-PML-RARα (anti-RARα or anti-HA), and GFP-SUMO5 in 293 cells was analyzed by confocal microscopy. (C) PML-RARα recruits SUMO1/2/3 into cytoplasmic aggregates. Subcellular localization of GFP-SUMOs and HA-PML-RARα in 293 cells was analyzed by confocal microscopy. (D) Endogenous SP100 partially co-localizes with SUMO5 in the nucleus in the presence of PML-RARα. 293 cells were transfected with GFP-tagged SUMO5 alone or cotransfected with GFP-SUMO5 and HA-PML-RARα. An antibody specific for SP100 was used to gauge the localization of endogenous SP100 in confocal microscopy. (E) HIPK2 partially co-localizes with SUMO5 in the nucleus in the presence of PML-RARα. 293 cells were transfected with combinations of plasmids indicated at the left of the panels. Subcellular localization of GFP-SUMO5 and Flag-HIPK2 was analyzed by confocal microscopy. (F) shSUMO5 specifically knocks down the expression of SUMO5. 293 cells were co-transfected with GFP-SUMO expression plasmids and the shRNA construct for SUMO5 or control shRNA (shNC). (G) Summary of the consequences of SUMO5 conjugation in pathological conditions such as APL. In the presence of PML-RARα, SUMO5 preferentially conjugates PML-RARα, resulting in decreased SUMO5 conjugation of PML. SUMO5-conjugated PML-RARα sequesters PML into the cytoplasm, leaving behind nuclear bodies without PML. Scale bars, 10 µm. (H) Summary of how SUMO5 regulates the PML-NB cycle. Mono-conjugation of PML by SUMO5, which is the seeding phase of PML-NB biogenesis, creates a platform upon which components of PML-NBs assemble through interaction and further SUMO5 conjugation. Enlargement of PML-NBs is facilitated by polySUMO5 conjugation of PML, which recruits other proteins into PML-NBs. Conjugation of SUMO2/3 and concomitant de-conjugation of SUMO5 from PML initiate the disruption process of PML-NBs.

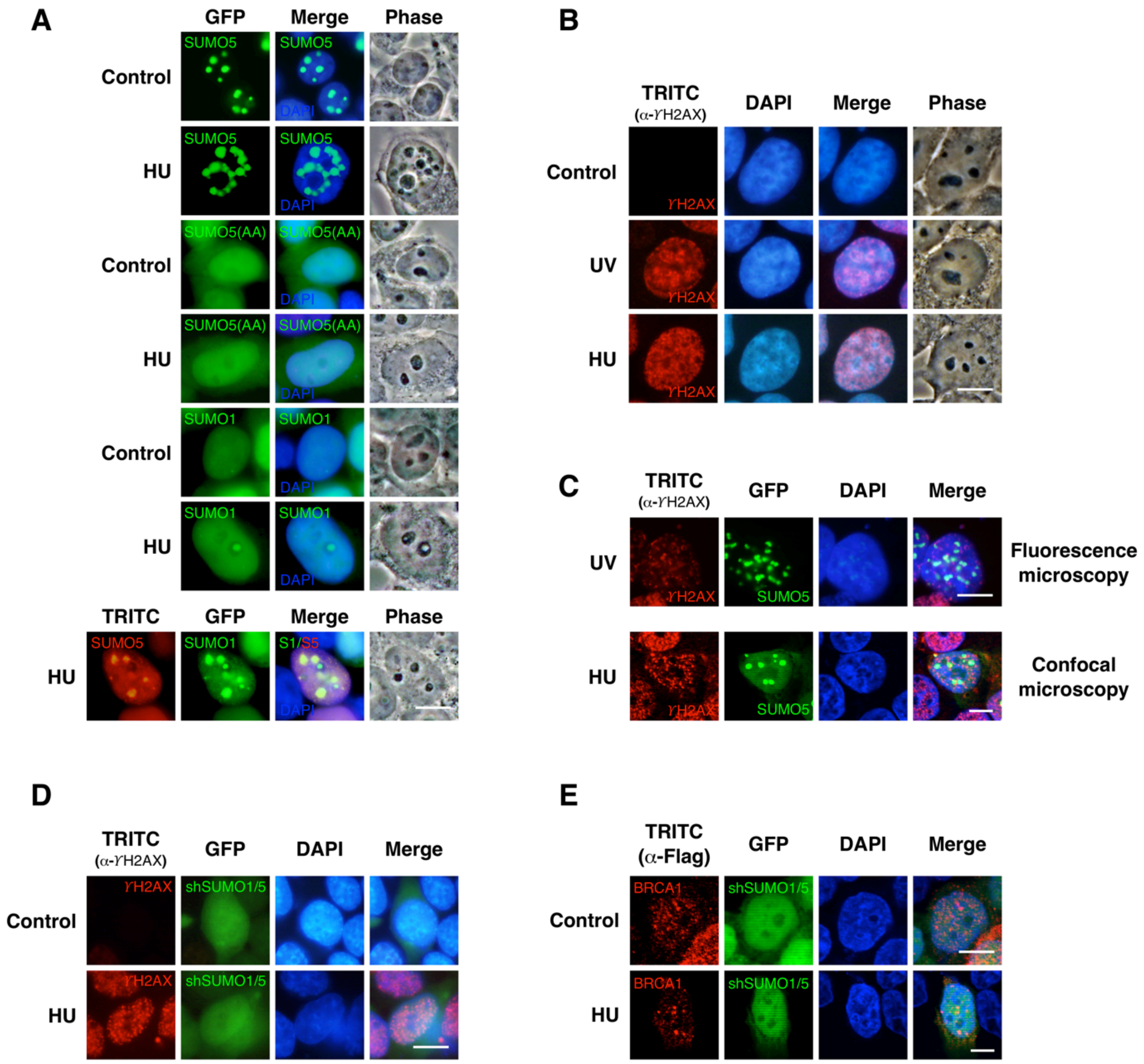

Figure S8. SUMO5-mediated nuclear bodies are involved in DNA damage response.

(A) Hydroxyurea (HU) induces the formation of SUMO5 nuclear bodies, which co-localize with DNA damage-induced SUMO1 foci except in the nucleolus. 293 cells were transfected with GFP-SUMO5, GFP-SUMO5(AA), GFP-SUMO1, or co-transfected with Flag-SUMO5 and GFP-SUMO1 expression plasmids. 24 hours post-transfection, cells were treated with water (control) or 3 µM of HU for 16 hours. Subcellular localization of SUMO5, SUMO5(AA), SUMO1 (green), and Flag-SUMO5 (red) was assessed by immunofluorescence microscopy with anti-Flag antibody or the GFP tag. DNA (blue) was stained with DAPI. Scale bars, 10 µm. (B) Treatment of 293 cells with 10 J/m^2 UVc and 3 µM of HU for 16 hours induced γH2AX signals. Cell images were obtained by fluorescence microscopy using anti-γH2AX antibody. (C) DNA damage-induced SUMO5 nuclear bodies are adjacent to γH2AX foci. 293 cells were transfected with GFP-SUMO5 expression plasmid. 24 hours post-transfection, cells were treated as described in (B). (D) Knockdown of SUMO1 and SUMO5 does not impair the formation of γH2AX foci. 293 cells were transfected with the SUMO1/5 shRNA construct (shSUMO1/5). 24 hours post-transfection, cells were treated with water (control) or 3 µM of HU for 16 hours. Cell images were obtained by fluorescence microscopy with anti-γH2AX antibody. (E) Knockdown of SUMO1 and SUMO5 decreases the formation of BRCA1 foci upon DNA damage. 293 cells were co-transfected with the SUMO1/5 shRNA construct (shSUMO1/5) and Flag-BRCA1 expression construct. 24 hours post-transfection, cells were treated with water (control) or 3 µM of HU for 16 hours. Cell images were obtained by confocal microscopy with anti-γH2AX or anti-Flag antibody. Images were representatives from multiple repeats of experiments.
